# Lipidation of Temporin-1CEb Derivatives as a Tool for Activity Improvement, Pros and Cons of the Approach

**DOI:** 10.3390/ijms22136679

**Published:** 2021-06-22

**Authors:** Paulina Kosikowska-Adamus, Emilia Sikorska, Dariusz Wyrzykowski, Aleksandra Walewska, Anna Golda, Milena Deptuła, Michał Obuchowski, Adam Prahl, Michał Pikuła, Adam Lesner

**Affiliations:** 1 Faculty of Chemistry, University of Gdansk, 80-309 Gdańsk, Poland; emilia.sikorska@ug.edu.pl (E.S.); dariusz.wyrzykowski@ug.edu.pl (D.W.); aleksandra.walewska@ug.edu.pl (A.W.); adam.prahl@ug.edu.pl (A.P.); adam.lesner@ug.edu.pl (A.L.); 2 Department of Microbiology, Faculty of Biochemistry, Biophysics and Biotechnology, Jagiellonian University, 30-387 Kraków, Poland; anna.b.golda@uj.edu.pl; 3 Laboratory of Tissue Engineering and Regenerative Medicine, Department of Embryology, Medical University of Gdansk, 80-210 Gdańsk, Poland; milenadeptula@gumed.edu.pl (M.D.); michal.pikula@gumed.edu.pl (M.P.); 4 Department of Medical Biotechnology, Intercollegiate Faculty of Biotechnology UG-MUG, Medical University of Gdansk, 80-210 Gdańsk, Poland; obuchowk@biotech.ug.edu.pl

**Keywords:** antimicrobial peptides, membranolytic properties of peptides, lipidation of peptides, mode of action of lipopeptides, AMP activity improvement

## Abstract

The alarming raise of multi-drug resistance among human microbial pathogens makes the development of novel therapeutics a priority task. In contrast to conventional antibiotics, antimicrobial peptides (AMPs), besides evoking a broad spectrum of activity against microorganisms, could offer additional benefits, such as the ability to neutralize toxins, modulate inflammatory response, eradicate bacterial and fungal biofilms or prevent their development. The latter properties are of special interest, as most antibiotics available on the market have limited ability to diffuse through rigid structures of biofilms. Lipidation of AMPs is considered as an effective approach for enhancement of their antimicrobial potential and in vivo stability; however, it could also have undesired impact on selectivity, solubility or the aggregation state of the modified peptides. In the present work, we describe the results of structural modifications of compounds designed based on cationic antimicrobial peptides DK5 and CAR-PEG-DK5, derivatized at their N-terminal part with fatty acids with different lengths of carbon chain. The proposed modifications substantially improved antimicrobial properties of the final compounds and their effectiveness in inhibition of biofilm development as well as eradication of pre-formed 24 h old biofilms of *Candida albicans* and *Staphylococcus aureus*. The most active compounds (C5-DK5, C12-DK5 and C12-CAR-PEG-DK5) were also potent against multi-drug resistant *Staphylococcus aureus USA300* strain and clinical isolates of *Pseudomonas aeruginosa*. Both experimental and in silico methods revealed strong correlation between the length of fatty acid attached to the peptides and their final membranolytic properties, tendency to self-assemble and cytotoxicity.

## 1. Introduction

The growing number of incidents of antibiotic resistance among pathogenic microorganisms represents one of the most serious healthcare challenges [1]. According to the latest report of World Health Organization, less than a third of the drugs that recently entered clinical trials represent a novel class of antibiotics with unique mechanisms of action [2]. A majority of the molecules in the pipeline are variants of established classes of antibiotics, such as derivatives of ß-lactam (carbapenem) in combination with diverse ß-lactamase inhibitors, fluoroquinolones, tetracyclines, oxazolidinones and macrolides [2,3]. Given that microorganisms tolerant to these classes of compounds already exist, their derivatives will be prone to class-based resistance mechanisms; thus, their long-term effectiveness could be substantially lowered. Therefore, there is an urgent need for compounds with both broad spectrum of antimicrobial activity and novel mechanisms of action [4]. In this context, we see the chance for peptide-based anti-infective drugs as a promising alternative to conventional antibiotics.

Antimicrobial peptides represent the group of endogenous compounds involved in the first-line immune response to pathogen invasion or tissue injury. They are produced by almost all living organisms (microorganisms, invertebrates, animal species or plants) and cover a structurally diverse group of molecules. A major proportion of currently identified AMPs is represented by short, cationic, amphipathic sequences with broad spectrum antimicrobial activity [5]. For a long time, AMPs were considered as electrostatically driven membranolytic agents. However, the most recent studies suggest, that their biological activity in vivo could be more complex and could also include immunomodulatory, endotoxin-neutralizing, anticancer, wound-healing or antibiofilm activity [6,7,8,9,10].

Recently, we have published results covering the synthesis of bicomponent peptide conjugates, which were composed of two different peptides with varying activities, i.e., pro-proliferative toward human skin cells (keratinocytes and fibroblasts) and exhibiting antimicrobial activity against bacterial and fungi strains. Within the scope of that project, we designed CAR-PEG-DK5, which contained naturally occurring endogenous dipeptide carnosine (CAR) and a synthetic analogue of amphibian temporin-1CEb (DK5) [11], respectively, bound together by means of chemically inert PEG-linker.

Carnosine was selected due to its established antioxidant, free radical scavenging, anti-inflammatory and wound-healing properties [12,13,14]. This dipeptide is widely distributed in muscles and nervous tissues, where it plays an important protective function [15]. In spite of a wide spectrum of biological functions, therapeutic use of free native carnosine is still limited due to its quick digestion by serum-circulating enzyme carnosinase. To overcome this problem, derivatization of its amino or carboxylic groups were undertaken [16]. In the present study, we utilize the N-terminal lipidation of carnosines and investigate their role as a component of a conjugate with antimicrobial peptide DK5.

The core of the conjugate, antimicrobial peptide DK5, is the chemical analogue of temporin-1CEb isolated from skin secretions of the North American and Eurasian frog *Rana amurensis*. This peptide was designed by the group of Shang and co-workers and was characterized as membranolytic with activity against both Gram-positive and Gram-negative bacteria [17]. Our previous studies showed that its covalent binding with carnosine yielded the compound with good antifungal (anti-*Candida* spps.) and moderate antibacterial properties and a stimulative effect on proliferation and migration of human keratinocytes and fibroblasts [11].

Here, we present the results of further structural modifications of DK5, CAR and CAR-PEG-DK5 by means of N-terminal lipidation with different type of carboxylic acids. Modification of peptides with lipophilic molecules is a well-known approach that enhances their membrane anchoring properties, and, as result, improves antimicrobial activity [18,19]. However, this strategy should be well considered in order to minimize the risk of cytotoxicity of the resulting lipopeptides toward mammalian cells. Previous studies suggest that the final effect of lipidation is difficult to predict as both lipophilic moieties attached to AMP and the peptide itself defines the final mode of action and spectrum of activity of the modified compounds [20]. Taking this into consideration, we performed both extensive in vitro screening of antimicrobial and cytotoxic properties of the modified AMPs, but also applied in silico methods, such as molecular dynamic simulations, or isothermal titration calorimetry (ITC) measurements, in order to analyze, in a multiplane way, all aspects of structure–activity relationship of this modification.

## 2. Results

### 2.1. Design, Synthesis and Purification of the Peptide Conjugates and Their Lipidated Derivatives

The previously characterized peptide DK5 and its conjugate CAR-PEG-DK5 were modified at their N-terminal fragment with valeric (C5), lauric (C12) and palmitic (C16) saturated fatty acids. Additionally, N-terminal acylation with 6-aminohexanoic acid (AHX) was performed. The same structural modifications were applied to carnosine. All designed compounds were obtained in high yields by means of a solid-phase method applying Fmoc/*tert-*butyl chemistry and were purified by means of a reversed phase HPLC approach to at least 98% of homogeneity (Figure S1, Supplementary Materials). Before introduction to in vitro biological tests, all compounds underwent TFA-counter-ion exchange through lyophilization from 0.1 M HCl solution [21]. All calculated and experimentally obtained physicochemical characteristics of the synthesized compounds are presented in Table 1.

### 2.2. CD Spectra Analysis

Relative content of α-helical structure within the sequences of the tested compounds was analyzed in phospholipid environment of POPG, which served as a simplified model of the anionic membrane. According to the obtained results, with characteristic negative bands at 208 nm and 220 nm, all compounds adopted α-helical conformation (84–100% of α-helicity) (Table 1; Figure S2, Supplementary Materials). Thus, N-terminal lipidation did not affect the tendency of conjugates and analogs of DK5 to form helical structures in POPG medium.

### 2.3. Antimicrobial Activity against Planktonic Forms of Microorganisms

In order to establish the spectrum of antimicrobial activity, compounds were tested against several Gram-negative, Gram-positive bacteria and pathogenic yeasts from *Candida* species. A broth microdilution method for determination of minimal inhibitory concentration (MIC) was used as a standard procedure. The results of the performed screening are presented in Table 2 and Table 3. According to the obtained data, all N-terminally modified analogues of DK5 were substantially more active than the parent peptide. The best results were observed for modification with lauric acid (C12-DK5), while the less distinct effect was obtained for the peptide acylated with palmitic acid (C16-DK5). In the case of analogs of CAR-PEG-DK5, only N-terminal introduction of lauric acid improved antimicrobial properties (C12-CAR-PEG-DK5). By contrast, lipidation of carnosine had no measurable antimicrobial effect. Moreover, analysis of the biological properties of these analogues was complicated due to their high hydrophobicity. Both C12-CAR and C16-CAR formed self-standing gels in water within the range of analyzed concentrations. According to Castelletto and co-workers [22], who performed a comprehensive study on C16-CAR, lipidation with palmitic acid stimulates self-assembly of the molecules in water into nanotapes able to interact with DPPC (dipalmitoylphosphatidylcholine) membranes causing their decomposition. As mentioned above, we observed that a water solution of C12-CAR (at a concertation of 1 mg/mL) underwent gelation as well. Taking into account similar volume constraints (in comparison to C16-CAR) imposed by lauric acid in relation to dipeptide, there is a high probability that C12-CAR lipopeptide could be prone to form nanofibres as well. In the case of C5-CAR and AHX-CAR, gelation in water was not observed at concentrations of up to 5 mg/mL; nevertheless, both derivatives were not active against tested microorganisms.

Based on the obtained results, we chose the most active compounds and evaluated their activity against multi-drug resistant *Sthaphylococcus aureus* USA300, a highly virulent strain that causes persistent and complicated skin and soft tissue infections and is able to colonize and survive inside the host cells, evading defense response [23,24]. Additionally, screening included two clinical isolates of *P*s*eudomonas aeruginosa* (PA1, PA2) and a reference strain of *Klebsiella pneumonia* (ATCC 13883) (Table 3). In comparison to the parent peptides (DK5 and CAR-PEG-DK5), which were inactive toward selected microorganisms at concentrations up to 200 µg/mL, their analogs effectively inhibited the growth of *S. aureus* USA300 with MICs ranging from 12.5 µg/mL (C12-CAR-PEG-DK5) to 25 µg/mL (C5-DK5, C12-DK5) and Gram-negative clinical isolates of PA1, PA2 and KP1 (MICs 25–50 µg/mL).

### 2.4. Viability of Bacteria in the Presence of the Lipopeptides

**Figure 1:**
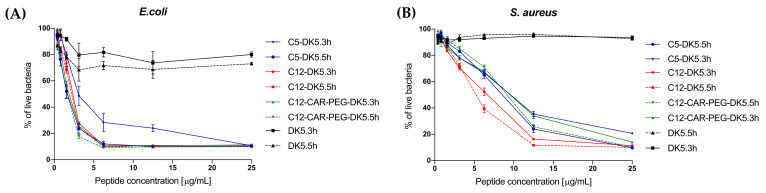
Analysis of the relative viability of *E. coli* (**A**) and *S. aureus* (**B**) suspensions in the presence of tested compounds after 3 and 6 h of co-incubation. The relative percentage of live bacteria was calculated from green/red fluorescence ratio for each concentration of the peptides. The data recorded for control probes (bacteria without peptide) incubated under the same conditions in the presence of both dyes (SYTO 9 and propidium iodide) were treated as 100%.

A live/dead bacterial differentiation in the presence of C5-DK5, C12-DK5, C12-CAR-PEG-DK5 and DK5, applied at different concentrations, was performed by means of BacLight^TM^ Bacterial Viability Kit. Two bacterial strains, *S. aureus* and *E. coli,* were used in order to see the effect on the microorganisms depending on their Gram stain status. All tested lipopeptides at concentrations around 10 µg/mL essentially eradicated *E. coli* within 3 h of co-incubation (Figure 1). Under the same experimental conditions, DK5 was found to be almost inactive against both strains. In the case of *S. aureus,* we observed more linear dependance between the concentration of the lipopeptides and their ability to compromise bacterial membranes. C12-DK5 was found to be the most active; it eradicated the bacteria approximately 20–30% better than its analogues C12-CAR-PEG-DK5 and C5-DK5.

### 2.5. Cytotoxic Properties towards Human Cells

**Figure 2:**
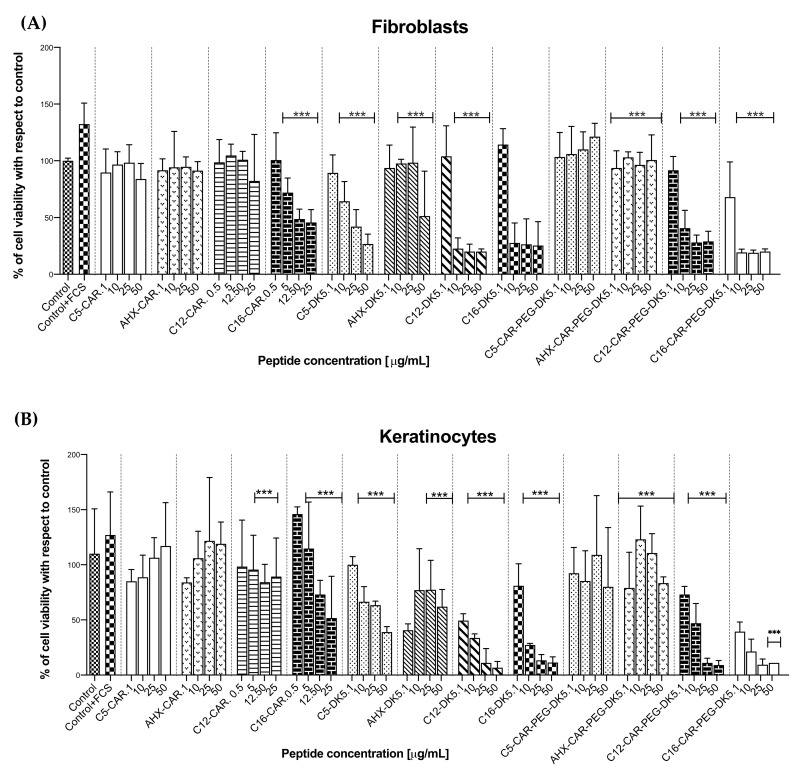
Evaluation of the cytotoxic effect of the tested compounds on human primary fibroblasts (**A**) and transformed cell line of human keratinocytes (**B**).The data cover the results of MTT colorimetric assay and express the percentage of cell viability of the certain probes with respect to control probe (untreated cells, incubated in serum-free DMEM). Additional positive control corresponds to cells incubated in the presence of 10% of FCS in DMEM. All data were obtained from three independent experiments performed in triplicates. Results are expressed with their standard deviation (error bars). *** *p* < 0.001 vs. control.

Cytotoxicity of the obtained peptides toward immortal human cell line of keratinocytes (HaCaT) and primary fibroblasts isolated from skin biopsies of healthy human was analyzed within a 24 h timeframe (Table 4; Figure 2). According to the results, lipidation of CAR-PEG-DK5 substantially affected its safety toward human cells. A clear negative correlation was observed between the length of the lipid moiety and cytotoxicity of the resulting compound. Both C5-DK5 and AHX-DK5 appeared to be moderately toxic at concentrations up to 50 µg/m towards both keratinocytes and fibroblasts. In turn, C5-CAR-PEG-DK5 and AHX-CAR-PEG-DK5 were neutral or even pro-proliferative toward both cell lines. In the case of compounds modified with lauric and palmitic acid, a significant cytotoxicity is observed at concentrations starting from 10 µg/mL. Conversely, lipidated carnosines generally did not affect cell viability, with the exception of C16-CAR, which caused a noticeable decline in cell viability at concentrations starting from 5 µg/mL. A slight positive dose-response correlation was found for C5-CAR and AHX-CAR towards keratinocytes; however, these lipopeptides remained neutral with respect to fibroblasts.

### 2.6. Antimicrobial Properties against Bacterial and Fungal Biofilms

In our studies, we focused on two aspects concerning biofilm: prevention of its development and dispersion/eradication of already formed biofilms. Antibiofilm properties were analyzed against *S. aureus* (PCM 2054) and *C. albicans*. Due to the lack of antimicrobial activity of lipidated carnosines, these compounds were excluded from antibiofilm screening. Taking into account the earlier established cytotoxicity of the compounds, we decided to analyze their effect within the same range of concentrations that were used in MIC screening.

The susceptibility of the bacterial and fungal biofilms to the designed compounds was analyzed by means of 3-(4,5-dimethylthiazol-2-yl)-2,5-diphenyltetrazolium bromide (MTT) reduction assay, as it enables identification of living cells within the complex structure of biofilms and does not interact with dead cells or components of the medium. Thus, this assay gives a reliable assessment of biofilm viability. BIC_50_ values were established after 24 h post-inoculation (Table 5, Figures S3–S6, Supplementary Materials). The best results were obtained for DK5 analogs against both *C. albicans* and *S. aureus*. In the case of conjugates, C12-CAR-PEG-DK5 and C16-CAR-PEG-DK5 effectively inhibited development of bacterial and fungi biofilms, while C5-CAR-PEG-DK5 and AHX-CAR-PEG-DK5 were almost inactive against them.

The reduction of the density of pre-formed 24 h old bacterial and fungal biofilms was analyzed in a similar manner. Surprisingly, we observed almost the same effectiveness of all lipidated compounds on pre-formed biofilm of *C. albicans* with an average BEC_50_ between 12.5 and 25 µg/mL_,_ with the exception of slightly more active C12-DK5 with a BEC_50_ of 6.25 µg/mL. In the case of *S. aureus* biofilm, the best effect was observed for C12-DK5 and C12-CAR-PEG-DK5 (BEC_50_ of 6.25 µg/mL), while the worse activities both in prevention of the development and in reduction of viability of pre-formed biofilms were observed for C5-CAR-PEG-DK5 and AHX-CAR-PEG-DK5. It should be mentioned that, within the selected range of concentrations from 50 to 6.25 µg/mL, none of the tested compounds completely eradicated bacterial or fungal biofilms. In contrast, the parent molecules, DK5 and CAR-PEG-DK5, were substantially less active than their lipidated analogs in both prevention of biofilm development and its eradication (Table 5; Figures S3–S6, Supplementary Materials).

### 2.7. Serum Stability

**Figure 3:**
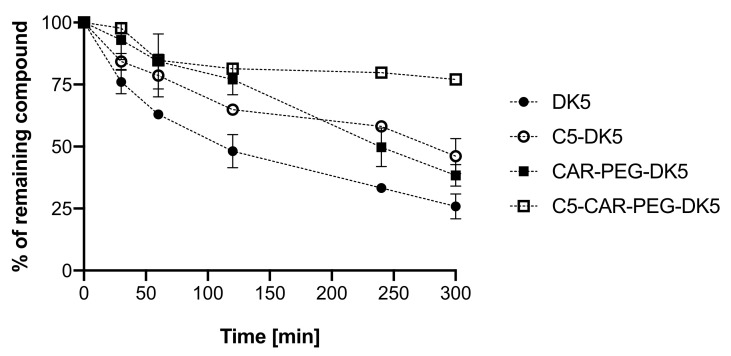
Evaluation of serum stability of the selected compounds.

In order to understand the influence of N-terminal acylation of the tested compounds on potential resistance to proteolytic degradation, we performed serum stability assay and also measured antimicrobial activity of the lipidated analogs in the presence of 1% human serum. Serum stability studies revealed that compounds acylated with valeric acid were substantially more stable after 5 h of incubation in human serum in comparison to their parent molecules (Figure 3). The amounts of the intact compounds at the end of the analysis were 46.2% and 77% for C5-DK5 and C5-CAR-PEG-DK5, respectively; while in the case of DK5 and CAR-PEG-DK5 within the same time frame, we obtained fractions containing 25.9 and 38.4% of the remaining compounds. The same experiments were performed for derivatives modified with palmitic and lauric acids. However, during incubation, we observed that these compounds spontaneously precipitated in water solution of serum; thus, it was disabled thorough separation of lipopeptide fractions and, hence, its adequate quantitative chromatographic analysis. Nevertheless, we expect that serum stability of these derivatives must be comparable to the results obtained for C5-analogs; however, their availability in the presence of serum is strongly affected due to both the tendency to auto-aggregate as well as binding to serum proteins and lipids.

**Figure 4:**
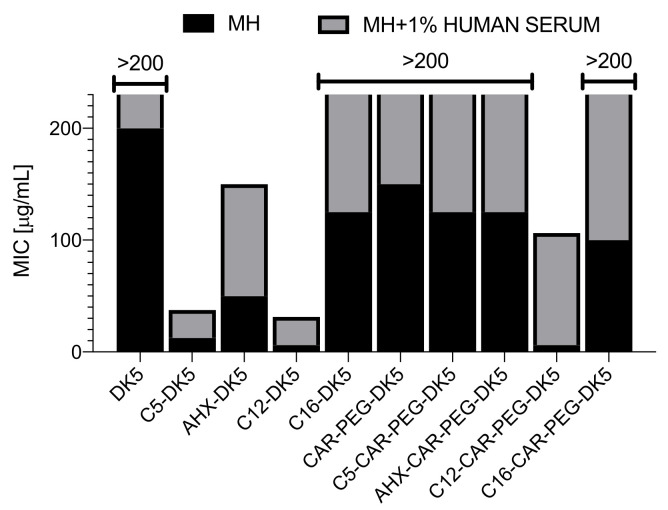
The comparative analysis of MICs established for *S. aureus* PCM 2054 strain in the presence or absence of 1% normal human serum. The black columns correspond to the results obtained in standard Mueller-Hinton broth, while the grey columns cover the results obtained in Mueller-Hinton broth supplemented with 1% of normal human serum.

These results were also confirmed during evaluation of MICs against *S. aureus* PCM 2054 strain in the presence of 1% human serum. Supplementation of growth medium with serum caused an almost complete reduction of the antimicrobial activity of CAR-PEG-DK5 and its lipidated analogs, while in the case of the most active DK5 derivatives, MICs increased from 12.5 to 25 µg/mL and from 6.25 to 25 µg/mL for C5-DK5 and C12-DK5, respectively (Figure 4).

### 2.8. Analysis of Interaction with Artificial Membranes by Means of ITC

#### 2.8.1. Peptide Binding to Anionic Membrane

**Figure 5:**
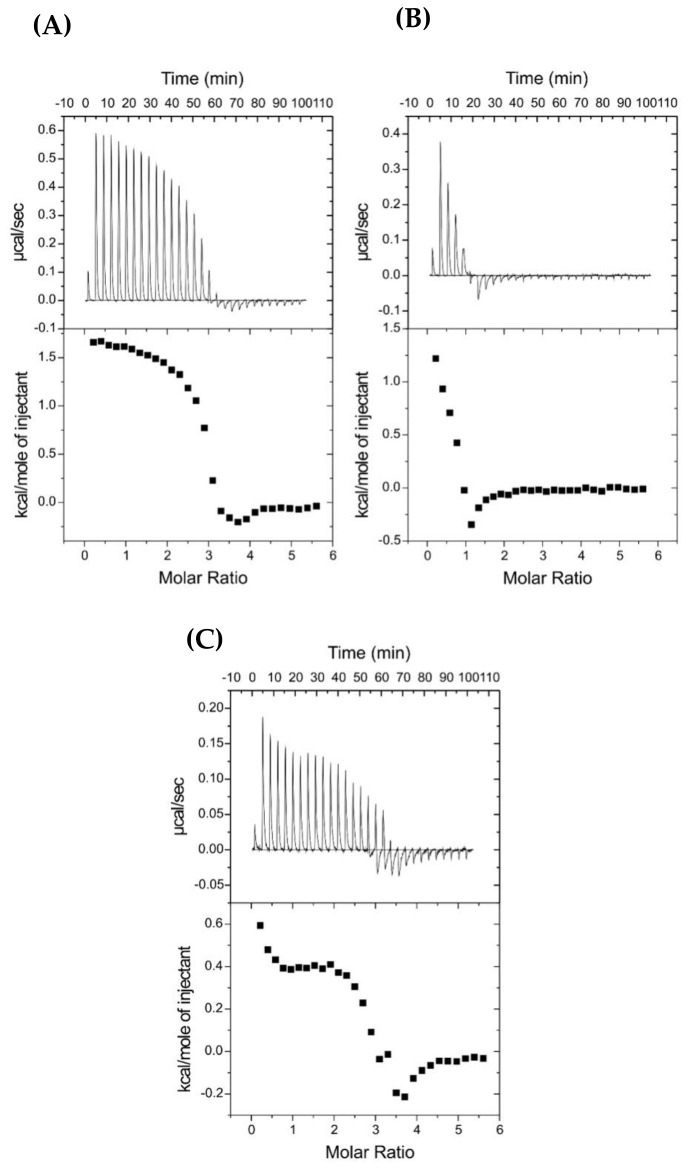
Isothermal calorimetric titration patterns obtained for titration of 0.05 mM (**A**) C5-DK5, (**B**) C12-DK5, (**C**) C12-CAR-PEG-DK5 solutions with 1.31 mM POPG LUVs at 25 °C. The lower curves represent the heat of reaction (measured by peak integration) as a function of the lipid/peptide molar ratio.

The three lipopeptides with the best profile of antimicrobial activity (C5-DK5, C12-DK5, C12-CAR-PEG-DK5) were selected for ITC analysis to characterize their possible mode of interaction with artificial membranes. Figure 5 shows the results of these experiments in which an anionic phospholipid vesicle suspension, POPG, was gradually injected into the reaction cell containing antimicrobial peptides. According to the obtained data, in the case of C5-DK5 and C12-DK5 (Figure 5A,B), the first injections produced an endothermic heat of reaction, which decreased in magnitude with successive injections. However, at L/P molar ratio ca. 3 and 1 for C5-DK5 and C12-DK5, respectively, small exothermic effects were observed, before saturation binding was achieved. An even more complex pattern was noticed for C12-CAR-PEG-DK5, where the heat of reaction no longer decreased smoothly with each injection, but showed inflection points, suggesting that the interaction between the peptides and POPG LUVs involves more than just binding. These results can be explained by other thermodynamic processes, such as pore formation, change in the lipid phase properties during the titration, micellization of phospholipid vesicles or initial peptide aggregation, superimposed on peptides partitioning into membranes [25,26,27]. Due to the superposition of the binding reaction with other unspecified processes, the binding parameters cannot be derived from ITC experiments, and only the quantitative conclusion can be made.

#### 2.8.2. Peptide Binding to a Zwitterionic Membrane

**Figure 6:**
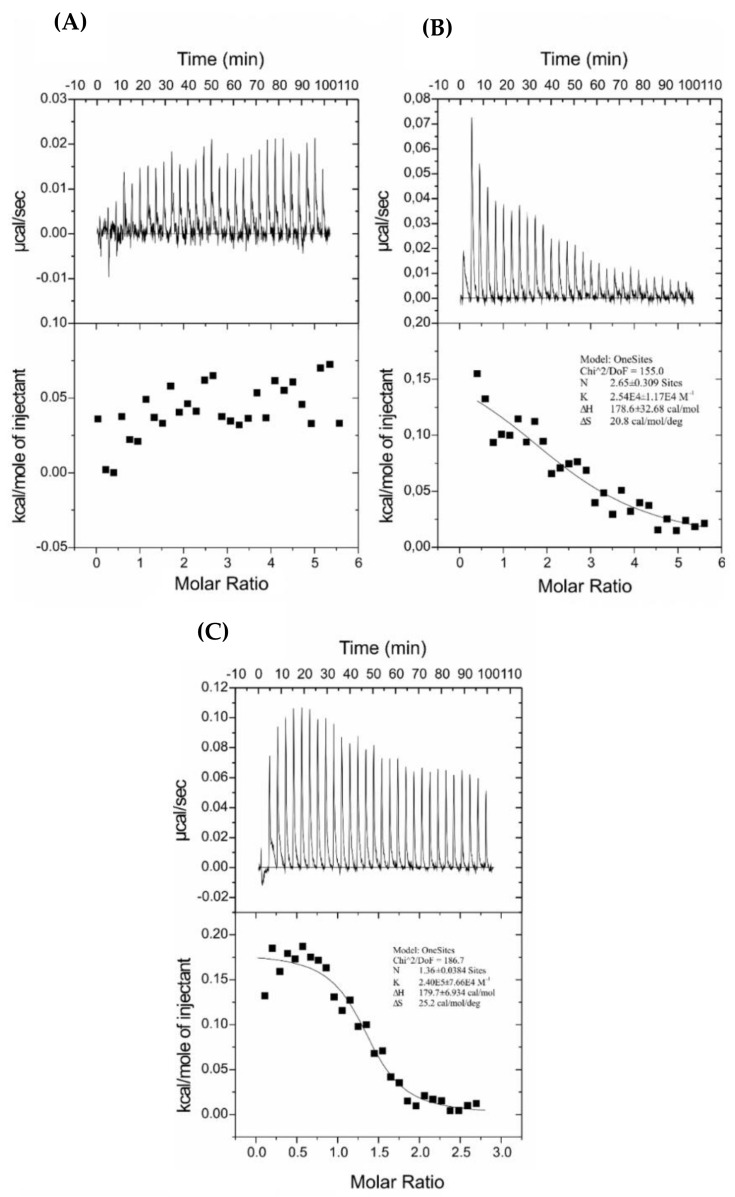
Isothermal calorimetric titration patterns obtained for titration of (**A**) 0.05 mM C5-DK5, (**B**) C12-DK5, (**C**) 0.1 mM C12-CAR-PEG-DK5 solutions with 1.31 mM POPC LUVs at 25 °C. The lower curves represent the heat of reaction (measured by peak integration) as a function of the lipid/peptide molar ratio.

The ITC traces obtained by titration of POPC LUVs into antimicrobial peptide solutions at 25 °C are shown in Figure 6. The thermodynamic parameters of the binding are collected in Table 6. Injections of the zwitterionic POPC lipids into the reaction cell with the studied peptides produced endothermic heats of reaction. However, in the case of C5-DK5, the titration indicates either a lack of interactions or that the binding was too weak to be detectable by ITC. The binding process of lauroyl-modified peptides to POPC vesicles resulted in the binding constants on the order of 10^−4^ and 10^−5^ M^−1^ for C12-DK5 and C12-CAR-PEG-DK5, respectively. The addition of the CAR-PEG fragment increased the binding constant by one order of magnitude, which reflects higher affinity of the C12-CAR-PEG-DK5 to zwitterionic lipids. Negative free energy indicates that, in both cases, binding process is spontaneous. The absolute value of TΔ***S*** is higher than that of Δ***H***, which means that the binding is an entropy-driven process.

### 2.9. Molecular Dynamic Simulations

**Figure 7:**
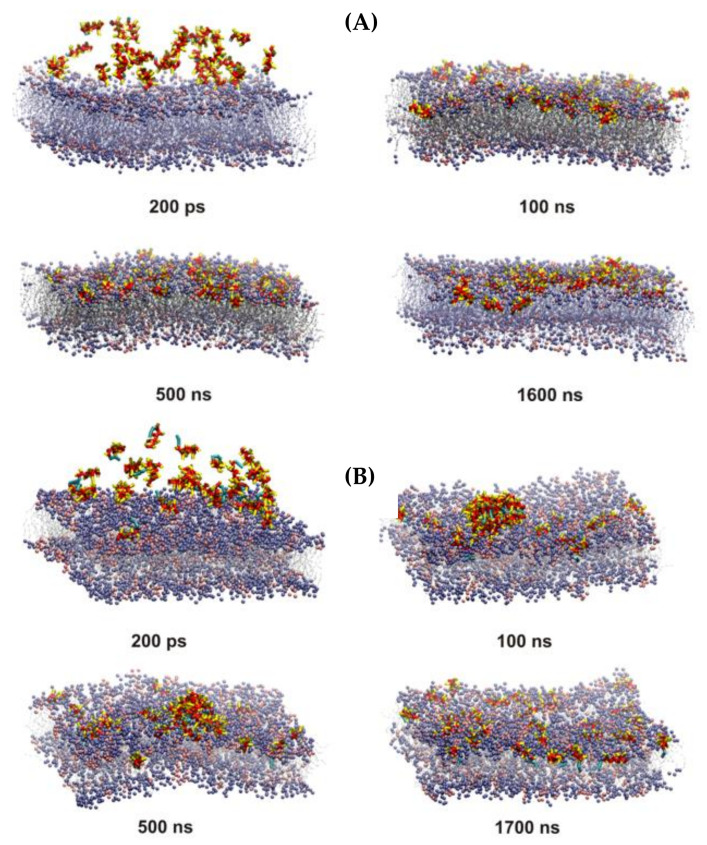
Snapshots from the 1:3 POPE:POPG binding simulations for C5-DK5 (**A**), C12-DK5 (**B**). Valeryl and lauroyl tails are colored cyan, and backbone and side chain beads are colored red and yellow, respectively. Lipid tails are colored gray, while lipid head groups are colored ice-blue and pink for POPG and POPE, respectively.

**Figure 8:**
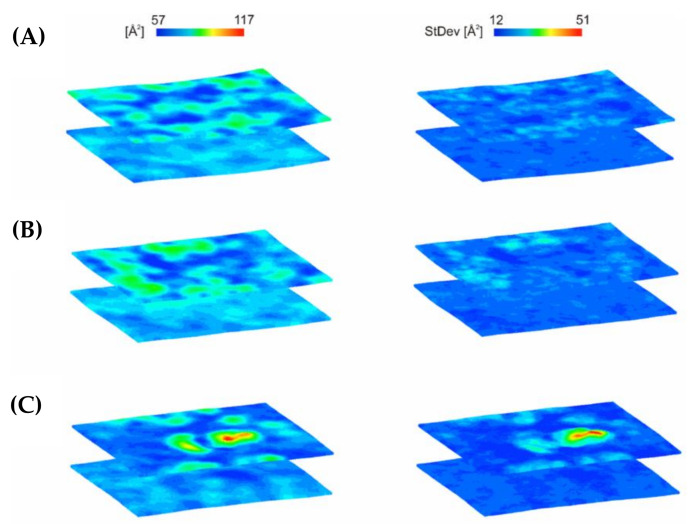
Local area per lipid (APL) of the 1:3 POPE:POPG bilayer averaged over last 100 ns of MD simulations for (**A**) C5-DK5, (**B**) C12-DK5 and (**C**) C12-CAR-PEG-DK5. Phosphate beads of the lipid headgroups were considered for calculations.

CG MD simulations were employed in order to analyze the possible dynamic of interaction of C5-DK5, C12-DK5 and C12-CAR-PEG-DK5 using a simplified membrane model. In the initial steps of the simulations, the association of the peptides with the bilayer surface occurred concurrently with the self-assembly process, with the exception of C5-DK5, for which self-assembly was not observed. C12-DK5 and C12-CAR-PEG-DK5 self-assembled into elongated micelles. Moreover, due to substantial crowding of the peptides at the beginning of the simulations and to the periodic boundary conditions, some peptide molecules were free to bind to the opposite leaflet of the lipid bilayer. These molecules were removed from the system within the first 100–200 ns of simulations in order to preserve the physiological conditions, in which only the outer face of the membrane is available for peptide binding. In the case of C12-DK5, the formed peptide aggregates were incorporated into the membrane in the subsequent steps of simulations. After insertion, the peptides dispersed throughout the membrane and, as expected for amphipathic peptide helices, they arranged with their helical axis parallel to the membrane surface (Figure 7). The peptide’s binding resulted in the local increase in the area per lipid (APL) in the outer leaflet of the membrane (Figure 8), which suggests disintegration of the membrane and can be related to the antimicrobial efficiency of the peptides.

**Figure 9:**
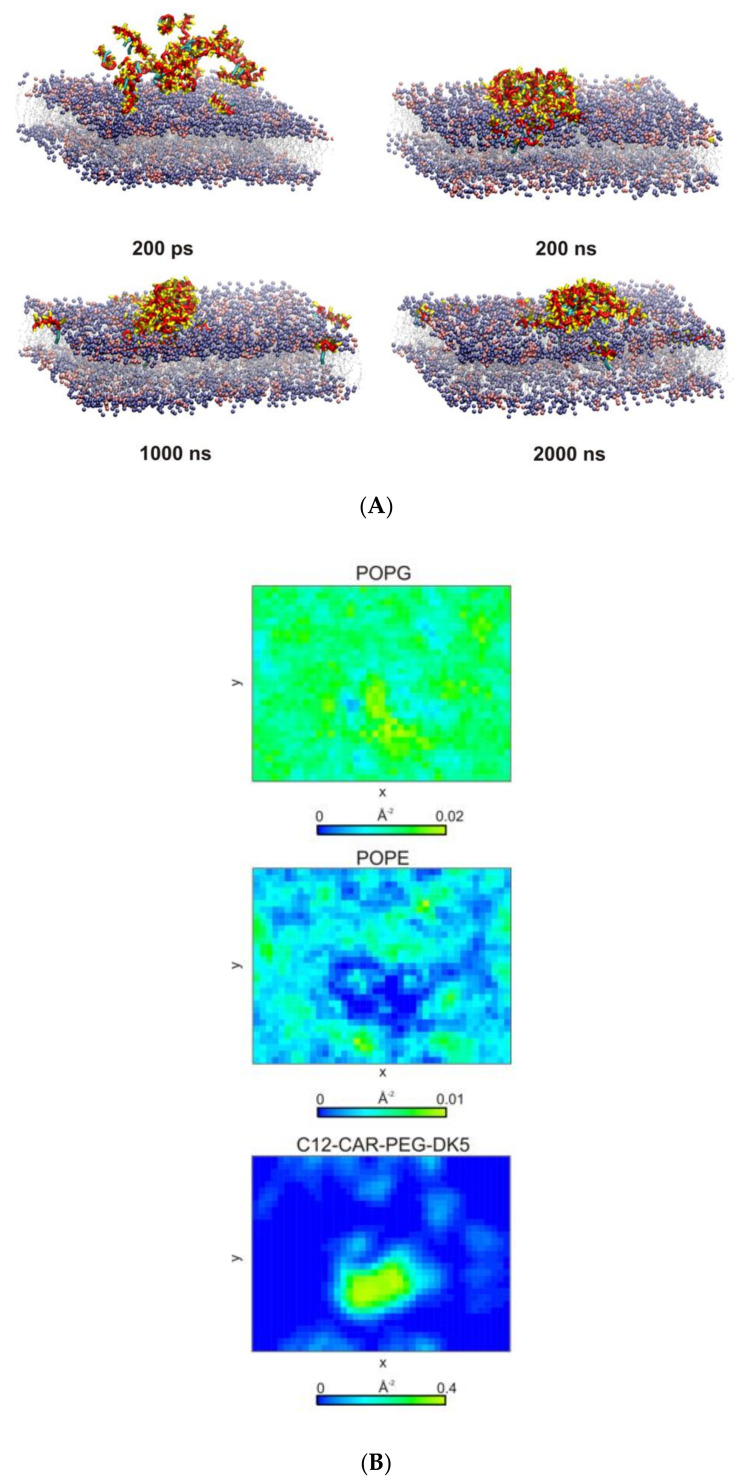

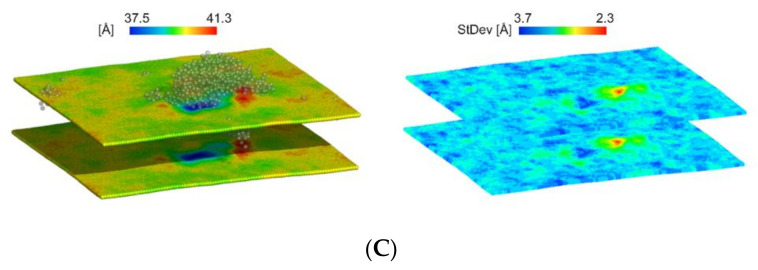
(**A**) Snapshots from the 1:3 POPE:POPG binding simulations for C12-CAR-PEG-DK5. Backbone and side chain beads are colored red and yellow, respectively. Lipid tails are colored gray, while lipid head groups are colored ice-blue and pink for POPG and POPE, respectively. (**B**) 2D density map of C12-CAR-PEG-DK5, POPG and POPE lipids in the upper leaflet of the membrane. A grid spacing was set to 5 Å. Last 100 ns of a total of 2 µs CG MD simulations were considered for analysis. (**C**) The time-averaged local thickness of the 1:3 POPE:POPG bilayer showing a perturbation of the membrane upon C12-CAR-PEG-DK5 binding. The data were averaged over last 100 ns of MD simulations. Phosphate beads of the lipid headgroups were considered for calculations.

With C12-CAR-PEG-DK5, an elongated micelle, formed at the beginning of the simulations, remained bound to the membrane surface even after 2 µs of simulations (Figure 9A). Nevertheless, it caused the formation of evident POPE-poor domain in the outer leaflet of the membrane in the space directly beneath, which indicates that the peptide changed the membrane lipid’s organization (Figure 9B). Additionally, the area per lipid at the micelle binding site deviates significantly from the optimal value. A noticeable increase in APL correlates with the thinning of the bilayer (Figure 8C and Figure 9C), which indicates that peptide adsorption can induce pore formation.

## 3. Discussion

In the present study, we analyzed the effect of N-terminal lipidation on antimicrobial and cytotoxic properties of the peptides CAR, DK5 and their chemical conjugate CAR-PEG-DK5. In the case of the modified carnosine, we observed that its acylation with valeric, aminohexanoic, lauric or palmitic acids did not induce antimicrobial activity of the resulting derivatives. Moreover, enhanced hydrophobicity of C12-CAR and C16-CAR caused their spontaneous self-assembling in water and promoted cytotoxicity toward human fibroblasts and keratinocytes.

In the case of the lipidated DK5 peptide and aforementioned CAR-PEG-DK5 conjugate, we obtained a group of analogs with diverse biological properties. CD spectra analysis confirmed that all lipopeptides retained their propensity to form helices upon membrane binding; therefore, this structural feature, often considered crucial for membranolytic activity of AMPs, was not affected. According to the results of antimicrobial susceptibility screening, C12-DK5 was the most active analog with about 10-fold improved activity against *C. albicans* and 20-fold against *C. glabrata* strains in comparison to the parent DK5 molecule. Moreover, C12-DK5 acquired antibiotic properties against *S. aureus* and *P. aeruginosa*, which were not evident for the DK5 peptide. Unfortunately, both C12-DK5 and C16-DK5 exerted high cytotoxicity against human skin cells. ITC measurements indicated that in addition to a complex mode of interaction with anionic POPG LUVs, C12-DK5 had a high affinity to zwitterionic lipids. The latter explains the observed drop in the selectivity of these compounds. Conversely, C5-DK5 lipopeptide modified with valeric acid was inert toward zwitterionic lipids. These findings correspond well to its moderate IC_50_ values toward human keratinocytes and fibroblasts. The C5-DK5 performed best among all DK5 derivatives, as it remained safe to human skin cells under its effective concentrations against broad spectrum of microorganisms, including methicillin-resistant *S. aureus* USA300 and clinical isolates of *P. aeruginosa*. It also remained relatively stable in human serum (about 50% of intact compound after 5 h of incubation in 25% serum). However, the highest tolerance in vitro to both cell lines used was observed for AHX-DK5 (IC_50_ > 50 µg/mL). This compound and the corresponding AHX-CAR-PEG-DK5 conjugate were synthesized as an example of lipidation that does not alter the charge of the molecule in comparison to the parent peptides. Our results showed that acylation with aminohexanoic acid improved antibacterial and antifungal properties of AHX-DK5 against selected *Candida* strains (with the exception of *C. krusei*); however, *B. cereus* and *S. aureus*, respectively, remained slightly sensitive or resistant to it.

In the case of conjugates, we observed a more differentiated activity profile: from almost inactive in the conjugates substituted with valeric and aminohexanoic acids, to moderately active in the derivative with N-terminal palmitate, with the analog modified with lauric acid exhibiting the best effect. ITC measurements performed for C12-CAR-PEG-DK5 showed that the presence of an N-terminal aliphatic C12-CAR- moiety promoted non-selective membrane targeting of this analog. C16-CAR-PEG-DK5, which might be subject to the same rule as both of these compounds, was cytotoxic toward mammalian cells. Additionally, molecular dynamic simulations revealed that the lipidated conjugate C12-CAR-PEG-DK5 had a strong tendency to self-assemble, which occurred concurrently with the process of membrane binding. In comparison to the results obtained for C5-DK5 and C12-DK5, which after initial binding diffused within the bilayer in a rather regular manner similar to the carpet model of membrane penetration, the C12 conjugate formed elongated micelles, and in such a solid bulky form, intruded into the phospholipid layers, forming the hydrophobic pore within. Our findings indicate that enhancement of lipophilicity of the N-terminally positioned CAR-PEG moiety by means of carboxylic acids with long carbon-tails negatively affects both antimicrobial properties and selectivity.

Serum stability studies confirmed that lipidated analogs were less prone to degradation in comparison to their parent molecules; however, the analogs of CAR-PEG-DK5 precipitated in the presence of serum, probably due to unspecific binding to serum proteins and lipids. The presence of 1% human serum in microbiological medium also negatively affected antimicrobial potency of all of the analyzed compounds against reference *S. aureus* strain. That could be explained by both partial proteolysis of the lipopetides and their co-aggregation with serum components, which is more apparent for generally stable CAR-PEG-DK5 analogs.

Antibiofilm activity studies showed that all N-derivatized analogs of DK5 effectively prevented biofilm formation of both *S. aureus* and *C. albicans* (average BIC_50_ ≤ 6.25 µg/mL); lipoconjugates were slightly weaker with the best activity established for C12-CAR-PEG-DK5 (BIC_50_ around 25 µg/mL). All of the analyzed compounds were able to eradicate the pre-formed biofilms to a certain extent, around 50% of killed microorganisms, but a clear dose-effectiveness response was not observed. The most probable reasons for the limited activity and plateau effect is the sequestration of the free floating lipopeptides by the binding to killed cells of microorganisms and dispersed fragments of biofilm, as well as their self-assembling at higher concentrations into micelles with restricted ability for biofilm penetration. However, it should be emphasized that, under the same experimental conditions, parent peptides DK5 and CAR-PEG-DK5 were almost inactive against biofilms. Again, considering the combination of antibiofilm properties and cytotoxicity, C5-DK5 stands as the most promising compound.

In summary, we conclude that the proposed modifications of the lead compounds DK5 and CAR-PEG-DK5 substantially improved the spectrum of antimicrobial activity and serum stability of the final derivatives. Additionally, these compounds became potent against pre-formed bacterial and fungal biofilms, and some of them were also able to reduce their development at concentrations close to their MICs. However, we also found that increased lipophilicity of the compounds with carboxylic acids of a longer carbon chain (lauric and palmitic acids) caused the reduction of selectivity and promoted self-aggregation of lipopeptides. The best result was achieved for the DK5 peptide acylated with valeric acid, while lipidation of conjugate CAR-PEG-DK5 facilitated its hydrophobicity and resulted in increased cytotoxicity combined with generally modest antimicrobial activity. All these findings suggest that the potential outcome of lipidation is defined both by the type of attached lipid moiety and physico-chemical properties of the modified peptide itself.

The observed increase in cytotoxicity of some of the obtained lipopeptides toward human skin cells represents the main drawback of the applied chemical modifications. However, in this case, we should also point out that the common problem of scientists working with AMPs arises from the lack of unified and reliable methods for testing their efficacy vs. safety. First of all, the outcome of the experiments is strongly correlated with the type of growth media, cell densities or inoculum concentrations, temperature and incubation conditions, etc. All these parameters differ greatly depending on the type of analyzed activity (antimicrobial, antibiofilm, anti-inflammatory or toxicity). Therefore, AMPs that appeared toxic under optimized conditions for cell culturing would not necessarily be harmful under more complex physiologically or clinically relevant conditions, and vice versa. These obstacles are discussed in detail in the interesting review published recently by Derry K. Mercer and co-authors [28], who emphasized the need for alternative methods of AMP activity assessment, as standard protocols developed for the purpose of laboratory diagnostics can significantly underestimate the therapeutic potential of the peptides. This problem was also mentioned by Savini and co-workers, who suggested the analysis of AMPs’ efficacy in co-cultures of bacterial and human cells as the more relevant model for simultaneous monitoring of both toxicity and antibiotic activity [28].

Thus, considering that no drug is completely safe and that further implementation of novel molecules is quite often a compromise between their toxicity and potential therapeutic benefits from their use, we see opportunity for the described lipopetides to serve as potential therapeutics of skin infections applied either alone or in synergistic combination with standard antibiotics.

## 4. Materials and Methods

### 4.1. Peptide Synthesis

All peptides and their lipidated derivatives were synthesized manually by means of the solid phase method applying Fmoc(fluorenyl-9methoxycarbonyl) chemistry under the standard conditions, as previously published [11]. S RAM (substitution 0.25 meq/g, RAPP Polymere, Germany) and 2-chlorotritylchloride (substitution 0.63 meq/g, GL Biochem, Shanghai Ltd.) resins were used as solid support. Lipidation of the peptides was performed dissolving the respective carboxylic acid (5 equiv.) in DMF/NMP (N-Methyl-2-pyrrolidone) and mixing it with HOBt/HBTU (5 equiv.). After that, DIPEA (10 equiv.) was added and the whole mixture was transferred to syringes containing the respective resins along with the immobilized peptide. The reaction was carried out overnight, after which the resins were washed with DMF and DCM (3 times both). The qualitative Kaiser test was used to monitor completeness of N-terminal lipidation. The resin-bound peptides and their lipidated derivatives were then cleaved from the resin with TFA/H_2_O/phenol/triisopropylsilane (88:5:5:2, *v/v/v/v*) mixture. After incubation, the resins were filtered out and the respective lipopeptides were isolated from the crude cleavage mixtures by precipitation with ice-cold diethyl ether and were further collected by centrifugation.

The obtained crude compounds were purified by reverse-phase high-performance chromatography (RP-HPLC) on Waters system (Phenomenex Jupiter 4 µ Proteo 90 Å column, 250 × 10 mm). The linear gradient from 10% to 80% B within 60 min (A: 0.1% TFA in water; B: 80% acetonitrile in A) with a flow rate 5 mL/min was employed. The homogeneity of the final fractions of the compounds were analyzed on a Shimadzu HPLC System (Shimadzu Europe GmbH, Duisburg, Germany) equipped with Phenomenex Jupiter 4 µ Proteo 90 Å column, 250 × 4.60 mm column. The identity of the final compounds were confirmed using of Biflex III MALDI TOF mass spectrometry (Bruker, Mannheim, Germany) with α-cyano-4-hydroxy-cinnamic acid (CCA) or 2,5-dihydroxybenzoic acid (DHB) used as the matrix protocol of the peptide synthesis.

### 4.2. Circular Dichroism

Circular dichroism spectra of the analyzed compounds were obtained by means of Jasco J-815 spectropolarimeter using a quartz cell of 1 mm path length between 195–260 nm at room temperature. Peptides at final concentration of 0.13 mM were mixed with 1.31 mM POPG solution (1-palmitoyl-2-oleoyl-sn-glycero-3-phosphoglycerol, Sigma-Aldrich, St. Louis, MO, USA) in phosphate buffered saline (PBS, 10 mM phosphate buffer containing 2.7 mM potassium chloride and 137 mM sodium chloride, pH 7.4). POPG vesicle system was used as the simplified model of negatively charged lipid membrane. On the basis of the obtained spectra, alpha-helical content was determined using a CONTINLL method implemented in CDPro software using, as a reference, the database of 43 soluble and 13 membrane bound proteins (SMP56) with precisely known secondary structures [29].

### 4.3. Antimicrobial Activity

#### 4.3.1. Determination of MIC

*Escherichia coli* (PCM 2057 = ATCC 25922), *Staphylococcus aureus* (PCM 2054 = ATCC 25923), *Staphylococcus epidermidis* (PCM 2118 = ATCC 14990), *Bacillus subtilis* (PCM 2224 = ATCC 9799), *Bacillus cereus* (PCM 482 = ATCC 10702) and *Pseudomonas aeruginosa* (PCM 499 = ATCC 10145) were obtained from the Polish Collection of Microorganisms. Fungi strains *Candida albicans* (CCM 8186 = CNTC 49/64), *Candida glabrata* (CCM 8270 = ATCC 90030), *Candida parapsilosis* (CCM 8260 = ATCC 22019) and *Candida krusei* (CCM 8271 = ATCC 6258) were acquired from the Czech Collection of Microorganisms. *S. aureus* USA300 (wild type strain), the reference strain of *Klebsiella pneumoniae* (ATCC 13883) and clinical isolates of *Pseudomonas aerugi*nosa (PA1, PA2) collected from nonrelated patients admitted to the Stefan Zeromski Specialist Municipal Hospital in Krakow (Poland) were kindly provided by the research group of the Department of Microbiology, Faculty of Biochemistry, Biophysics and Biotechnology, Jagiellonian University, Krakow, Poland.

Luria-Bertani broth (LB) and Sabouraud Dextrose broth (SB) (Biocorp, Poland) were used for cultivation of bacteria and fungi strains, respectively. The antimicrobial activity of the peptides were analyzed through determination of MIC (minimal inhibitory concentration) parameters according to Clinical and Laboratory Standards (CLSI) guidelines developed for aerobic bacteria [30] and yeasts [31], respectively. Mueller-Hinton broth (MHB) was used as the working medium for all bacterial strains, while in the case of fungi, RPMI-1640 (with L-glutamine, without sodium bicarbonate) growth medium buffered with MOPS (morpholinepropanesulfonic acid) and supplemented with 2% glucose was used. Inoculum was prepared from freshly grown cultures of microorganisms at their exponential phase of growth. Each well of 96-well plates containing 100 μL of serially diluted peptides in sterile water at concentration ranging from 100 to 6.25 μg/mL were inoculated with 100 μL of approximately 10^5^ CFU/mL of bacteria or about 2.5 × 10^3^ CFU/mL of fungi suspended in a double concentrated Mueller-Hinton or RPMI-1640 broth (supplemented with 2% glucose for *Candida* spps.), respectively. Plates were then incubated for 24 h at 37 °C in the case of bacteria and at 35 °C in the case of fungi. After that time, absorbance was read at 590 nm with a Perkin Elmer plate reader. Cultures without peptide were treated as positive control, while uninoculated media were defined as negative control. All measurements were run at least in triplicate. MIC is defined as the minimal concentration expressed in µg/mL, which completely inhibited visible growth of microorganisms.

Evaluation of MICs against *S. aureus* PCM 2054 strain in the presence of 1% human serum was performed as described above, but bacterial growth medium Mueller-Hinton broth was supplemented with 1% human serum (human serum, from human male AB plasma, Sigma-Aldrich).

#### 4.3.2. Biofilm Inhibition Assay

In the case of bacteria, an overnight culture of *S. aureus* in Tryptic Soy Broth (TSB) supplemented with 2% glucose (TSB/+G) was used to prepare inoculum for the experiments. Briefly, 5 mL of 24 h culture was centrifuged at 5000× *g*, washed with sterile PBS and suspended in a fresh 2 × concentrated TSB/+G. In the case of yeasts, the overnight culture of *C. albicans* in Sabouraud dextrose broth (SB) was used for inoculum preparation. Similar to above, 5 mL of 24 h culture was centrifuged, washed with PBS and then suspended in sterile 2 × concentrated RPMI-1640 medium (with L-glutamine, without sodium bicarbonate) supplemented with 2% glucose (RPMI-1640/+G). In both cases, the cell concentration of the inoculum was about 1 × 10^7^ cells per ml.

For the determination of the inhibitory concentration that reduced biofilm development by 50% (BIC_50_^,^ expressed in µg/mL), 100 µL inoculum in 2 × concentrated RPMI-1640/+G medium (in the case of *C. albicans*) or 2 x concentrated TSB/+G broth (in the case of *S. aureus*) was transferred to each well of 96-well plates containing 100 µL of serially diluted in water tested compounds. The final concentrations of the compounds were the same as those used in MIC determination studies. The 96-well plates were then incubated for 24 h at 37 °C and 35 °C in the case of bacteria and fungi, respectively. Viability of the microorganisms in the presence of the tested compounds was analyzed by means of MTT assay.

#### 4.3.3. Biofilm Eradication Assay

Biofilm eradication activity of the synthesized compounds was analyzed against 24 h old biofilms of *S. aureus* and *C. albicans*. In the case of bacteria, an overnight culture of *S. aureus* in Tryptic Soy Broth (TSB) supplemented with 2% glucose (TSB/+G) was used to prepare inoculum for the experiments. Briefly, 5 mL of 24 h culture was centrifuged at 5000× *g*, washed with sterile PBS and suspended in a fresh TSB/+G. The bacterial suspension was then diluted in a fresh growth medium to obtain the final concentration of colonies of about 1 × 10^7^ cells per ml. Further, 200 μL of the inoculum was transferred to each well of 96-well sterile polystyrene flat-bottomed plate, and bacteria were then incubated for the next 24 h without shaking at 37 °C.

In the case of yeasts, the overnight culture of *C. albicans* in Sabouraud dextrose broth (SB) was used for inoculum preparation. Similar to above, 5 mL of 24 h culture was centrifuged, washed with PBS and then suspended in sterile RPMI-1640 medium (with L-glutamine, without sodium bicarbonate) supplemented with 2% glucose (RPMI-1640/+G) yielding the amount of about 1 × 10^7^ cells per ml. Subsequently, 200 µL of yeast suspension was transferred into each well of a microtiter plate, and it was incubated stationary for the next 24 h at 35 °C.

After that, each well of the plates containing bacterial or fungi biofilm was washed three times with PBS in order to remove all non-adherent cells. Then, peptide solutions of 200 µL ranging from 50 to 0,39 µg/mL prepared in TSB +/G in the case of experiments on *S. aureus* biofilm, and (RPMI-1640/+G) in the case of *C. alibcans*, were prepared and transferred into the microtiter plates. After the addition of the peptides, the experiments were continued for the next 24 h.

The reduction in biofilm viability was analyzed by means of MTT assay. Briefly, 20 μL of MTT solution (5 mg/mL in PBS) was added to prewashed wells with exchanged medium (180 μL of fresh medium) wells containing bacterial/yeast biofilms. Plates were then incubated in the dark for the next 3 h at 37 °C. After that time, the medium was replaced with 6 mM HCl in isopropanol and, when the reduced MTT was fully dissolved, the plates were read at 570 nm. Activity against fully developed 24 h old biofilm was described as biofilm eradication concentration (BEC_50_ expressed in µg/mL), which corresponded to the concentration of the compound able to reduce 50% of biofilm viability compared with the growth control.

#### 4.3.4. Assessment of Bacterial Viability by Means of LIVE/DEAD BacLight Kit

Evaluation of the bacterial viability in the presence or absence of the tested compounds was performed according to the protocols developed for the laboratory kit LIVE/DEAD ^®®^ BacLight TM Bacterial Viability Kits (Molecular Probes Europe BV, Amsterdam, The Netherlands). Briefly, late log phase cultures of *E. coli* and *S. aureus* in Luria-Bertani broth were harvested by centrifugation at 4000× *g*, the supernatant was removed, and pellets were resuspended in sterile water solution of 0.85% NaCl. Again, bacterial cells were centrifuged under the same conditions. In order to remove traces of growth, the medium washing step was repeated twice. At the end, pellets were resuspended in a fresh sterile 0.85% NaCl solution and diluted, yielding suspensions of *E. coli*, containing about 2 × 10^8^ CFU/mL, and *S. aureus* with about 2 × 10^7^ CFU/mL. Subsequently, 90 µL of bacterial suspensions (*E. coli* or *S. aureus*, respectively) were transferred to 96-well microtiter plates and 10 µL of the tested peptides were added to final concentrations ranging from 0.39 µg/mL to 25 µg/mL. At the last step, 100 µL of 0.85% NaCl solution containing 0.6 µL of 3.34 mM SYTO9 dye and 0.6 µL of 20 mM propidium iodide was added to each well. Fluorescence was read with excitation wavelength set at 485 nm and emission wavelengths set at 530 nm (for green dye) and 630 nm (red dye) by means of CLARIOstar^®®^ plate reader (BMG LABTECH, Germany).

### 4.4. Cell Culturing Conditions

Human dermal fibroblasts were obtained as previously reported [11] from biopsies of healthy human skin of three donors undergoing a surgery. All procedures were approved by the Ethics Committee of Medicinal University of Gdansk (NKEBN/483/2011 and NKBBN/745/2019-2020). Immortalized human HaCaT keratinocytes (DKFZ Heidelberg, Germany) [32] were obtained commercially.

Both types of cell lines were grown in Dulbecco′s Modified Eagle′s Medium (DMEM) (Sigma-Aldrich, Germany), containing 4500 mg/mL of glucose, 584 mg/mL of L- glutamine, sodium pyruvate and sodium bicarbonate. Additionally, the medium was supplemented with 10% FCS (fetal calf serum, Sigma-Aldrich, Germany), 100 U/mL of penicillin and 100 μg/mL of streptomycin (Sigma-Aldrich, Germany). The cells were grown in standard culture dishes (BD, USA) in a humidified atmosphere with 5% CO2 at 37% C, and the medium was changed every 2 days.

### 4.5. Evaluation of Cytotoxicity towards Human Cells

The fibroblasts and keratinocytes were seeded at density of 5500 cells per well into 96-well plates suspended in a medium supplemented with 10% FCS. After 24 h from seeding, the media were exchanged with serum- and antibiotic-free DMEM and tested peptides, dissolved in sterile water, which were added, yielding a final concentration ranging from 1 to 50 μg/mL. The cells were then incubated for the next 24 h. At the end of the experiment, the cells were treated with 20 μL of MTT (5 mg/mL in PBS). After 3 h of incubation in the presence of MTT dye, the DMEM medium was discarded and replaced with 6 mM HCl in isopropanol. When the purple crystals of formazan were fully solubilized, absorbance at 570 nm was read.

### 4.6. Serum Stability Studies

At the beginning of the experiment, 25% human serum (human serum, from human male AB plasma, Sigma-Aldrich) was centrifuged at 12,000 rpm for 10 min in order to remove the excessive amount of lipids in the serum preparation, and the supernatant was collected and incubated at 37 °C for 15 min under shaking. The assay was initiated upon the addition of the peptide to the serum yielding a final peptide concentration of 75 µg/mL. The 50 µL aliquots of the incubated mixtures were taken at the following time intervals: 0, 30, 60, 120, 240 and 300 min. The aliquots were mixed with 50 µL of absolute ethanol, incubated on ice for 15 min to precipitate serum proteins and then centrifuged (12,000 rpm, 10 min). The supernatants were collected and lyophilized. Dry samples were further dissolved in 100 µL of HPLC-grade water and then analyzed. As a control, we used 25% human serum treated the same way and collected under the same time intervals.

RP-UPLC analysis was performed using Nexera system (Shimadzu Europe GmbH, Duisburg, Germany) with C12 Jupiter Proteo column (150 × 2 mm, 90Å, 4 micron) (Phenomenex, USA) applying a linear aqueous acetonitrile gradient 1–80%B in 20 min using solutions A (0.1% TFA in water) and B (0.1%TFA in 80% acetonitrile) with a flow rate 0.6 mL/min and detection at 220 nm. The quantity of the intact peptide at each time point was expressed as a percentage of the peak area with respect to time “zero” (100%).

Additionally, we performed a comparative analysis of MICs obtained for the tested compounds against *S. aureus* PCM2054 in two types of growth medium, i.e., the standard MHB and MHB supplemented with 1% human serum and in the absence of 1% serum. The assay was performed as described in 4.3.1.

### 4.7. Statistical Analysis

Statistical analysis was performed using GraphPad Prism 5 software according to one-way ANOVA analysis for repeated measurements applying post hoc Tukey’s multiply comparison test with significance level alpha set as 0.05 (*p* < 0.05).

### 4.8. ITC Measurements

The ITC experiments were performed according to the previously reported protocol [33] in PBS (pH 7.4) at 25 °C using an AutoITC isothermal titration calorimeter (MicroCal Inc., Northampton, USA) with a 1.4491-mL sample and reference cells filled with distilled water. In each experiment, 29 injections of 10.02 µL each (2 µL for the first only) of a liposome solution at a concentration of 1.31 mM were added into a 0.05 mM and/or 0.1 mM peptide solution. An initial 2-μL injection was discarded from each dataset in order to remove the effect of titrant diffusion across the syringe tip during the equilibration process. Each injection lasted 20 s. The titrant was injected at 4 min intervals to ensure that the titration peak returned to the baseline before the next injection. To ensure a homogeneous mixing in the cell, the stirrer speed was kept constant at 300 rpm.

A complete binding of the peptide to lipid vesicles provides the possibility for determination of the total binding enthalpy (∆H) as well as a binding constant (Ka), reflecting the affinity of the lipopeptide to liposomes, stoichiometry of binding (*n*), that is, the number of phospholipids per peptide, by fitting the titration result data to one set of sites’ model using Origin 7 from MicroCal. Changes in entropy (∆S) and the Gibbs free energy (∆G) were calculated from Equation (1), where factor 55.5 is the molar concentration of water, R is the gas constant (1.986 cal·mol^−1^·K^−1^) and T is the absolute temperature:

∆G = -RTln(55.5K_a_) = ∆H-T∆S (1)

The liposomes for ITC experiments were made of POPC or POPG lipids. The lipids were dissolved in a chloroform:methanol (2:1; *v*/*v*) mixture, evaporated under nitrogen and desiccated under vacuum overnight to remove any residual solvents. Afterwards, the dry lipid film was resuspended in a phosphate buffer solution and shaken for 2 h at room temperature (phase transition point, Tm = −2 °C) to obtain multilamellar vesicles (MLVs) [34]. Finally, the MLV suspension was extruded through polycarbonate membranes (100 nm in diameter, Whatman International Ltd., Dorset, UK) using a mini-extruder (Avanti Polar Lipids, Inc., Alabaster, AL, USA) to obtain large unilamellar vesicles (LUVs) used in titration experiments.

### 4.9. Molecular Dynamic Simulations

Simulations were carried out using the GROMACS 4.6.3 package [35]. The MARTINI coarse-grained force field [36,37] was used to explore the peptide-membrane interactions. The membrane was built of POPE and POPG at a molar ratio of 1:3 to mimic the membrane of Gram-positive bacteria [38]. The system was built using *insane.py* script available on the martini website (available online: http://cgmartini.nl (accessed on 25 June 2015). A total of 986 lipids were split and equally distributed between two membrane leaflets. Thirty molecules of each peptide were placed randomly on one leaflet of the membrane. The initial structure of each peptide was built with an α-helix in DK5 fragment. To preserve a helical structure during the MD simulations, the standard Martini topology was extended with extra harmonic bonds between non-bonded beads based on a distance cut-off. Each system was solvated and neutralized by sodium and chloride ions. The concentration of free salt ions was ca. 100 mM. Simulations were carried out for 1.6–2 µs in the isothermal-isobaric (NPT) ensemble with semi-isotropic pressure of 1 bar and at a constant temperature of 303 K. Statistical and trajectory analysis of the CG MD simulations were performed with the utilities included in GROMACS package and g_lomepro software [38], while visualizations were made with VMD [39] and PYMOL (The PyMOL Molecular Graphics System, Version 2.0 Schrödinger, LLC.).

## Figures and Tables

**Table 1:** Primary structures of the compounds with their basic physicochemical characteristics.

No	Compound	Primary Sequence	MW calc./obs.	Net Charge	α-Helicity [%]	RT [min]
1	C5-CAR	CH_3_(CH_2_)_3_C(O)-βAH	309.4/311.4	+0.1	-	10.67
2	AHX-CAR	NH_2_(CH_2_)_5_C(O)-βAH	339.4/340.2	+1.01	-	6.80
3	C12-CAR	CH_3_(CH_2_)_10_C(O)-βAH	407.6/409.5	+0.1	-	27.09
4	C16-CAR	CH_3_(CH_2_)_14_C(O)-βAH	462.7/463.5	+0.1	-	33.47
5	C5-DK5	CH_3_(CH_2_)_3_C(O)-IKKILS*K*IKKLL-NH_2_	1508.0/1508.6	+5	89.6	24.46
6	AHX-DK5	NH_2_(CH_2_)_5_C(O)-IKKILS*K*IKKLL-NH_2_	1537.0/1537.7	+6	85.6	20.53
7	C12-DK5	CH_3_(CH_2_)_10_C(O)-IKKILS*K*IKKLL-NH_2_	1606.2/1607.3	+5	83.7	30.53
8	C16-DK5	CH_3_(CH_2_)_14_C(O)-IKKILS*K*IKKLL-NH_2_	1662.32/1663.5	+5	100	34.25
9	C5-CAR-PEG-DK5	CH_3_(CH_2_)_3_C(O)-βAH-PEG- IKKILS*K*IKKLL-NH_2_	1861.4/1863.2	+5.1	94.7	21.39
10	AHX-CAR-PEG-DK5	NH_2_(CH_2_)_5_C(O)-βAH-PEG- IKKILS*K*IKKLL-NH_2_	1890.5/1892.3	+6.1	87.5	19.70
11	C12-CAR-PEG-DK5	CH_3_(CH_2_)_10_C(O)-βAH-PEG- IKKILS*K*IKKLL-NH_2_	1959.6/1961.6	+5.1	86.3	25.96
12	C16-CAR-PEG-DK5	CH_3_(CH_2_)_14_C(O)-βAH-PEG- IKKILS*K*IKKLL-NH_2_	2015.7/2017.0	+5.1	85.2	28.90

**Table 2:** The summarized results of antimicrobial properties of the tested lipopeptides.

Compound	MIC_100_ [µg/mL]									
	*C.albicans* *CCM 8186*	*C.krusei* *CCM 8271*	*C.parapsilosis* *CCM 8260*	*C.glabrata* *CCM 8270*	*B. subtilis* *PCM 2224*	*B. cereus* *PCM 482*	*S. epidermidis* *PCM 2118*	*S. aureus* *PCM 2054*	*E. coli* *PCM 2057*	*P. aeruginosa* *PCM 499*
C5-CAR	Non-inhibitory									
AHX-CAR										
C12-CAR										
C16-CAR										
C5-DK5	6.25	25	3.13	6.25	0.78	12.5	0.78	12.5	3.13	25
AHX-DK5	12.5	50	12.5	12.5	0.78	50	0.78	>50	12.5	25
C12-DK5	3.13	3.13	3.13	3.13	0.78	12.5	0.78	6.25	12.5	12.5
C16-DK5	>50	6.25	12,5	>50	3.13	>50	25	>50	>50	>50
C5-CAR-PEG-DK5	>50	>50	50	>50	6.25	>50	6.25	>50	>50	>50
AHX-CAR-PEG-DK5	>50	>50	50	>50	6.25	>50	6.25	>50	>50	>50
C12-CAR-PEG-DK5	12.5	12.5	3.13	12.5	3.13	>50	0.78	6.25	3.13	12.5
C16-CAR-PEG-DK5	25	12.5	6.25	>50	12.5	>50	25	>50	>50	>50
DK5	31.3	7.8	31.3	62.5	15.6	>50	7.8	>50	>50	50
CAR-PEG-DK5	15.6	3.9	31.3	62.5	3.9	>50	3.9	>50	>50	50

**Table 3:** Antibacterial properties of the selected compounds against clinical isolates of *P. aeruginosa*, *K. pneumonia* and MRSA strains.

Compound	MIC _100_ [µg/mL]			
	*K.pneumoniae* ATCC 13883	*P. aeruginosa* PA1	*P. aeruginosa* PA2	*S. aureus* USA300
DK5	>200	100	100	>200
CAR-PEG-DK5	>200	100	100	>200
C5-DK5	50	25	25	25
C12-DK5	25	50	50	25
C12-CAR-PEG-DK5	12.5	12.5	25	12.5

**Table 4:** The calculated IC_50_ values established for the tested compounds toward keratinocytes (HaCaT) and primary fibroblasts’ cell lines.

	IC_50_ [µg/mL]													
	C5-CAR	AHX-CAR	C12-CAR	C16-CAR	C5-DK5	AHX-DK5	C12-DK5	C16-DK5	C5-CAR-PEG-DK5	AHX-CAR-PEG-DK5	C12-CAR-PEG-DK5	C16-CAR-PEG-DK5	DK5	CAR-PEG-DK5
Keratinocytes	>50	>50	>50	25	35	>50	1	5	>50	>50	10	1	>50	>50
Fibroblasts	>50	>50	>50	12.5	20	50	7	7	>50	>50	12.5	4	>50	35

**Table 5:** The calculated values of BIC_50_ and BEC_50_ established against *S. aureus* and *C. albicans* biofilms.

	BIC_50_ [µg/mL]		BEC_50_ [µg/mL]	
	*S. aureus*	*C. albicans*	*S. aureus*	*C. albicans*
C5-CAR	Not determined			
AHX-CAR				
C12-CAR				
C16-CAR				
C5-DK5	6.25	10	>50	12.5
AHX-DK5	6.25	25	25	25
C12-DK5	6.25	6.25	6.25	1.56
C16-DK5	6.25	>50	6.25	12.5
C5-CAR-PEG-DK5	>50	>50	>50	20
AHX-CAR-PEG-DK5	>50	>50	>50	20
C12-CAR-PEG-DK5	20	10	20	12.5
C16-CAR-PEG-DK5	10	25	6.25	15
DK5	>50	>50	>50	>50
CAR-PEG-DK5	>50	50	>50	>50

**Table 6:** Thermodynamic parameters of binding C12-DK5 and C12-CAR-PEG-DK5 peptides to POPC LUVs at 25 °C.

Peptide	*n*	p*K*_ITC_	Δ*H* [kcal mol^−1^]	TΔ*S* [kcal mol^−1^]	Δ*G* [kcal mol^−1^]
C12-DK5	2.65 ± 0.31	4.40 ± 0.19	0.18 ± 0.03	8.56 ± 0.26	−8.38 ± 0.26
C12-CAR-PEG-DK5	1.36 ± 0.04	5.38 ± 0.14	0.18 ± 0.01	9.89 ± 0.19	−9.71 ± 0.19

## Data Availability

Not applicable.

## Supplementary Materials

The following are available online at https://www.mdpi.com/article/10.3390/ijms22136679/s1, Figure S1: Reverse Phase High Performance Liquid Chromatography (RT-HPLC) analysis performed for the purified fractions of the lipopeptides, including (A) C5-CAR, (B) AHX-CAR, (C) C12-CAR, (D)C16-CAR; (E) C5-DK5, (F) AHX-DK5, (G) C12-DK5, (H) C16-DK5, (I) C5-CAR-PEG-DK5, (J) AHX-CAR-PEG-DK5, (K) C12-CAR-PEG-DK5, (L) C16-CAR-PEG-DK5; Figure S2: CD spectra of the selected lipopetides obtained in POPG liposome medium; Figure S3: Inhibition of *S. aureus* biofilm formation by the tested lipopeptides; Figure S4: Inhibition of *C. albicans* biofilm formation by the tested lipopeptides; Figure S5: Eradication of the pre-formed 24-h old biofilm of *S. aureus* after 24-h treatment with the tested lipopeptides; Figure S6: Eradication of the pre-formed 24-h old biofilm of *C. albicans* after 24-h treatment with the tested lipopeptides.
