# Supplementary material for: Lipidation of Temporin-1CEb Derivatives as a Tool for Activity Improvement, Pros and Cons of the Approach
Source: Int J Mol Sci. 2021 Jun 22;22(13):6679. doi: 10.3390/ijms22136679 (PMC8269107; doi:10.3390/ijms22136679)
Supplement: Supplementary file 1 [file ijms-22-06679-s001.zip › ijms-1237033-supplementary.pdf]

Supplementary Materials:

(A)

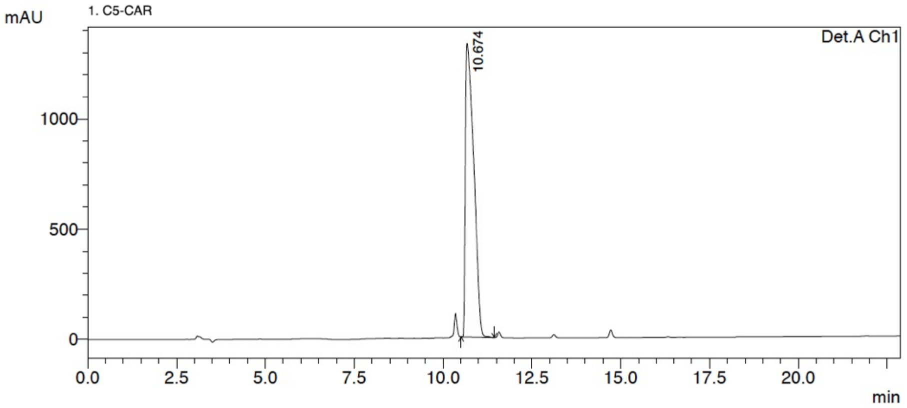

(B)

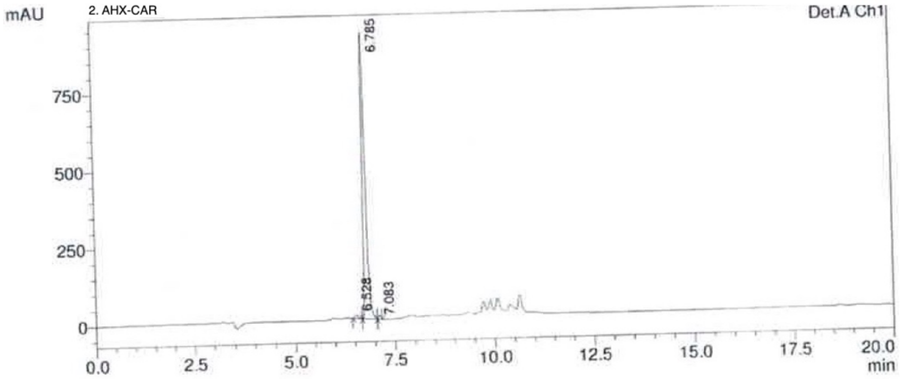

(C)

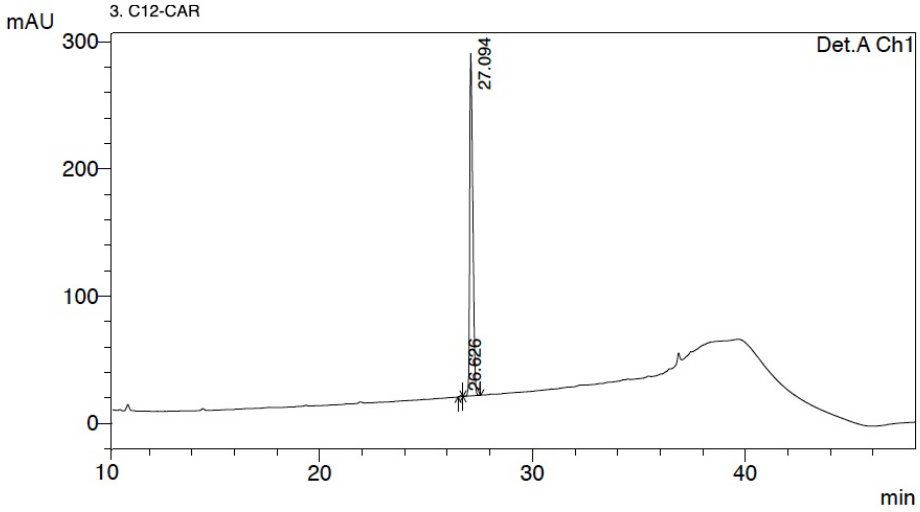

(D)

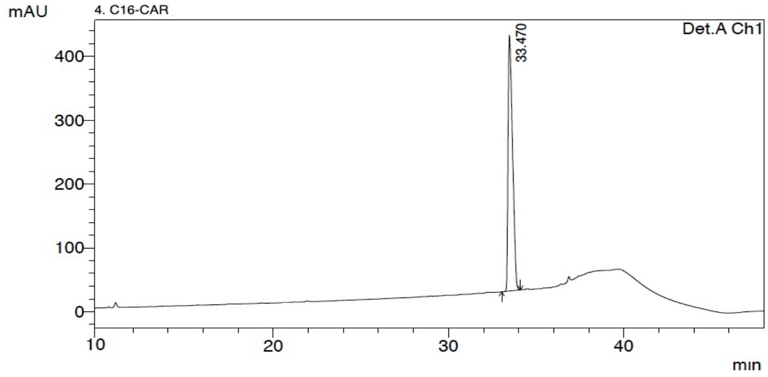
mAU
4. C16-CAR
Det.A Ch1
33.470
min

(E)

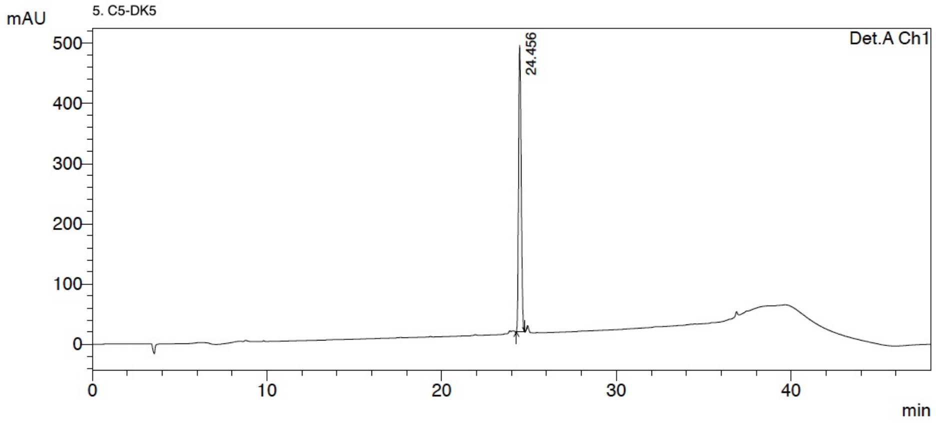
mAU
5. C5-DK5
Det.A Ch1
24.456
min

(F)

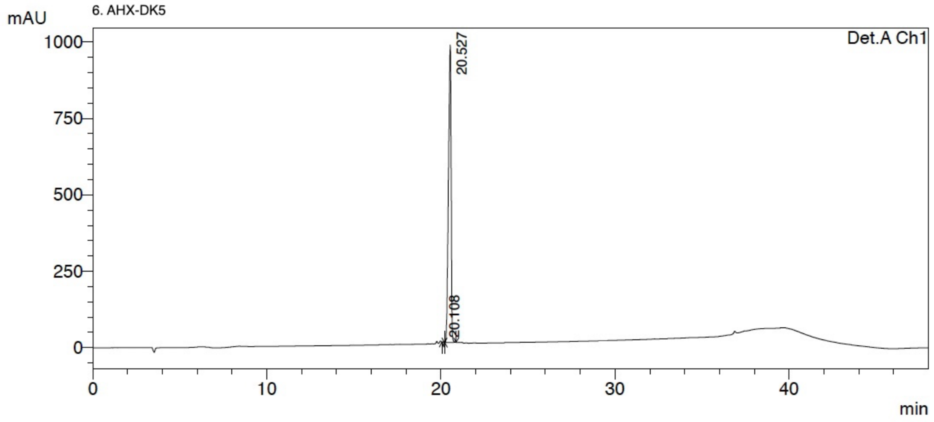
mAU
6. AHX-DK5
Det.A Ch1
20.527
20.108
min

(G)

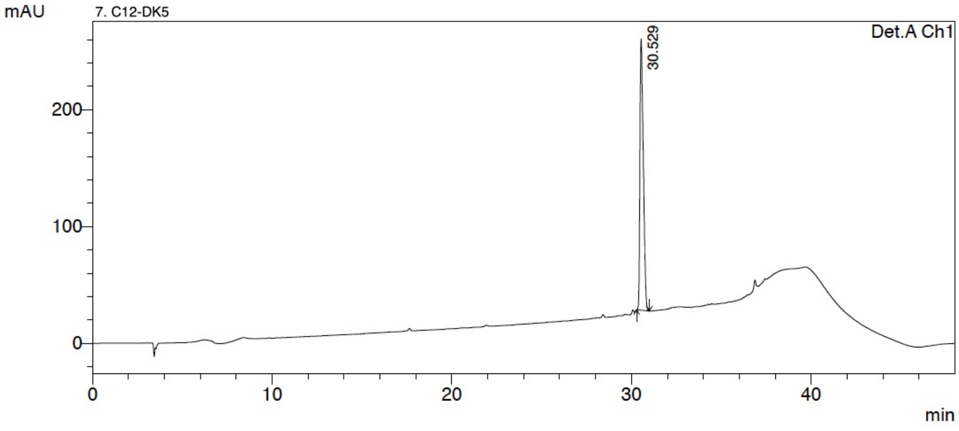
mAU
7. C12-DK5
Det.A Ch1
30.529
min

(H)

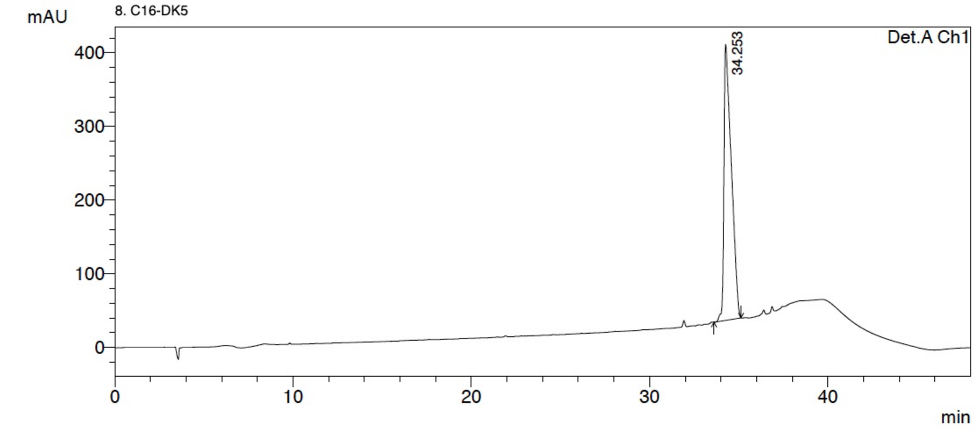
mAU
8. C16-DK5
Det.A Ch1
34.253
400
300
200
100
0
0
10
20
30
40
min

(I)

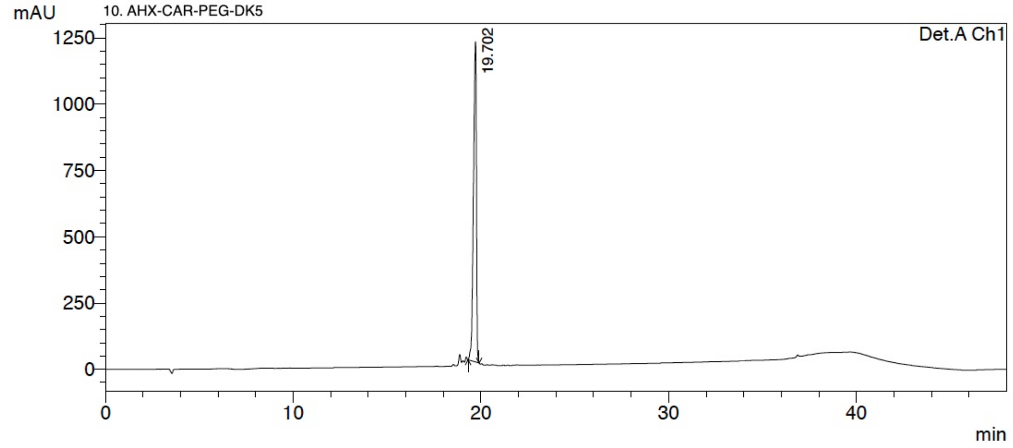
mAU
10. AHX-CAR-PEG-DK5
Det.A Ch1
19.702
1250
1000
750
500
250
0
0
10
20
30
40
min

(J)

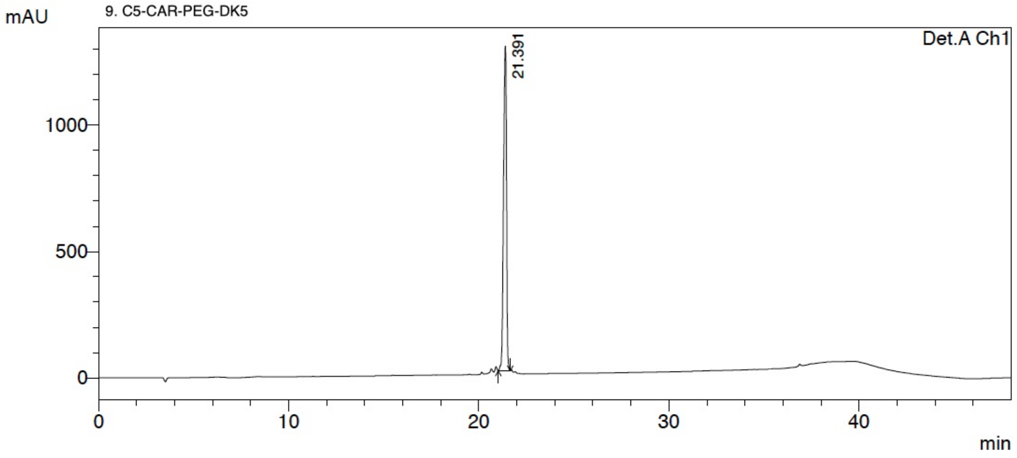
mAU
9. C5-CAR-PEG-DK5
Det.A Ch1
21.391
1000
500
0
0
10
20
30
40
min

(K)

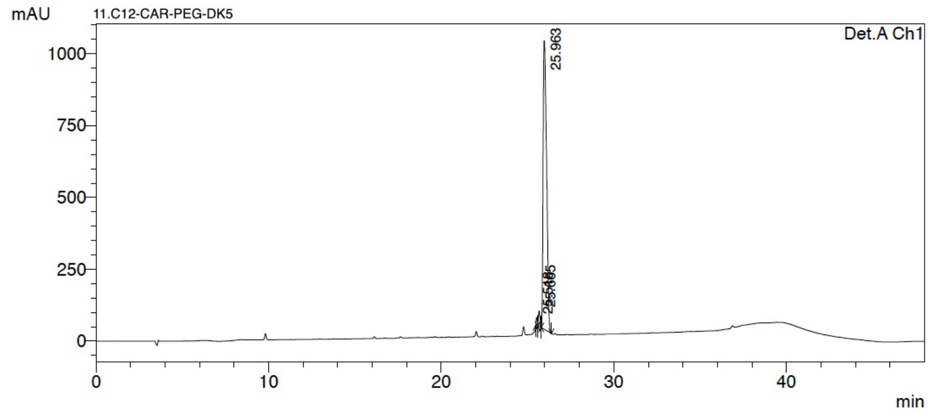

(L)

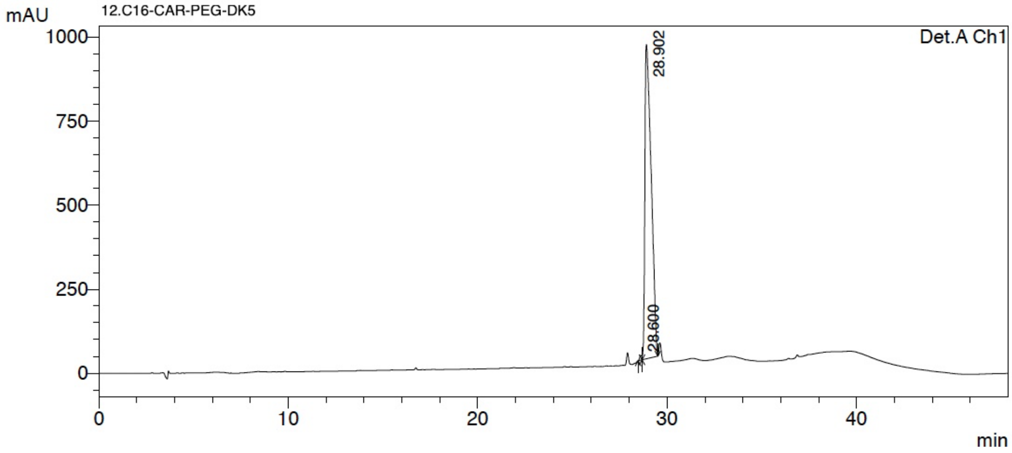

Figure S1. Reverse Phase High Performance Liquid Chromatography (RT-HPLC) analysis performed for the purified fractions of the lipopeptides, including **(A)** C5-CAR, (**B**) AHX-CAR, (**C**) C12-CAR, (**D**) C16-CAR; (**E**) C5-DK5, (**F**) AHX-DK5, (**G**) C12-DK5, (**H**) C16-DK5, (**I**) C5-CAR-PEG-DK5, (**J**) AHX-CAR-PEG-DK5, (**K**) C12-CAR-PEG-DK5, (**L**) C16-CAR-PEG-DK5.

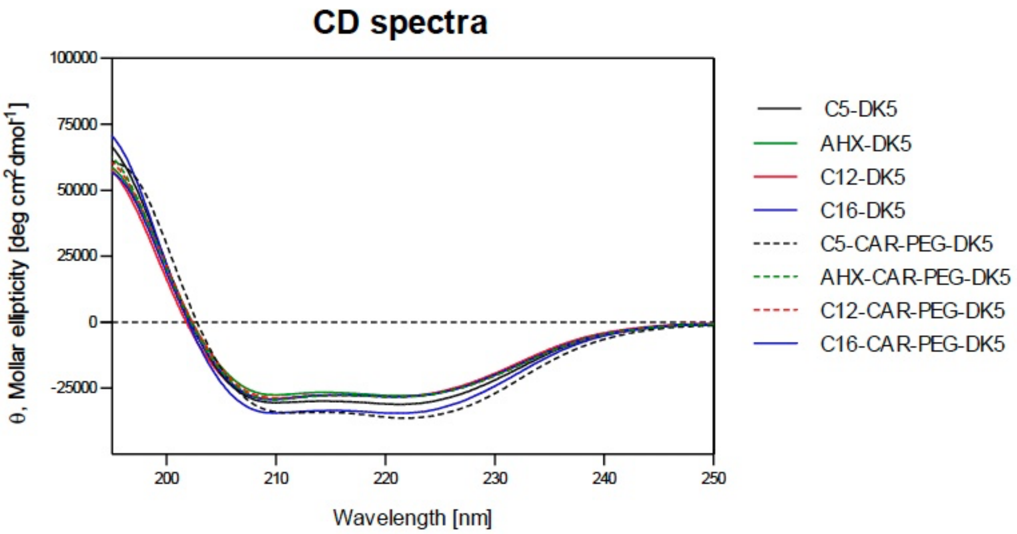

Figure S2. CD spectra of the selected lipopeptides obtained in POPG liposome medium.

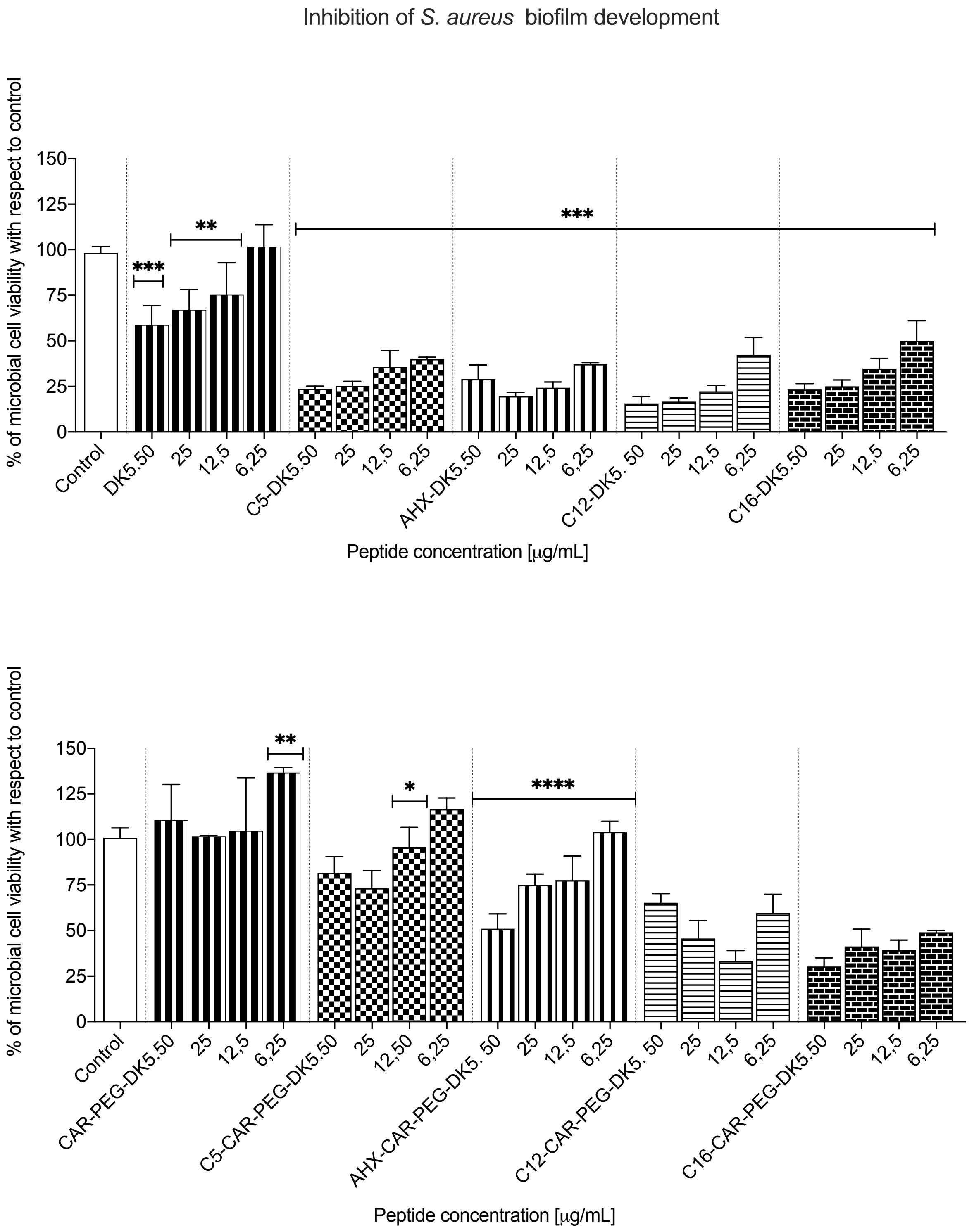

Figure S3. Inhibition of *Staphylococcus aureus* biofilm formation by the tested lipopeptides. The antibiofilm activity of the compounds against *S. aureus* (PCM 2054) strain was determined after 24 hours of incubation of the microorganisms in the presence of the selected lipopeptides. The data represents results of MTT assay performed at the end of the experiment. Microbial cell viability within pre-formed biofilms is expressed as the percentage of the cell viability of certain probes with respect to control (microorganisms incubated without peptides). The results were obtained from three independent experiments performed in triplicates and are expressed with standard deviation (error bars).* $P < 0.01$, **$P < 0.005$, ***$P < 0.0005$, ****$P < 0.0001$ vs. control

Inhibition of *C. albicans* biofilm development

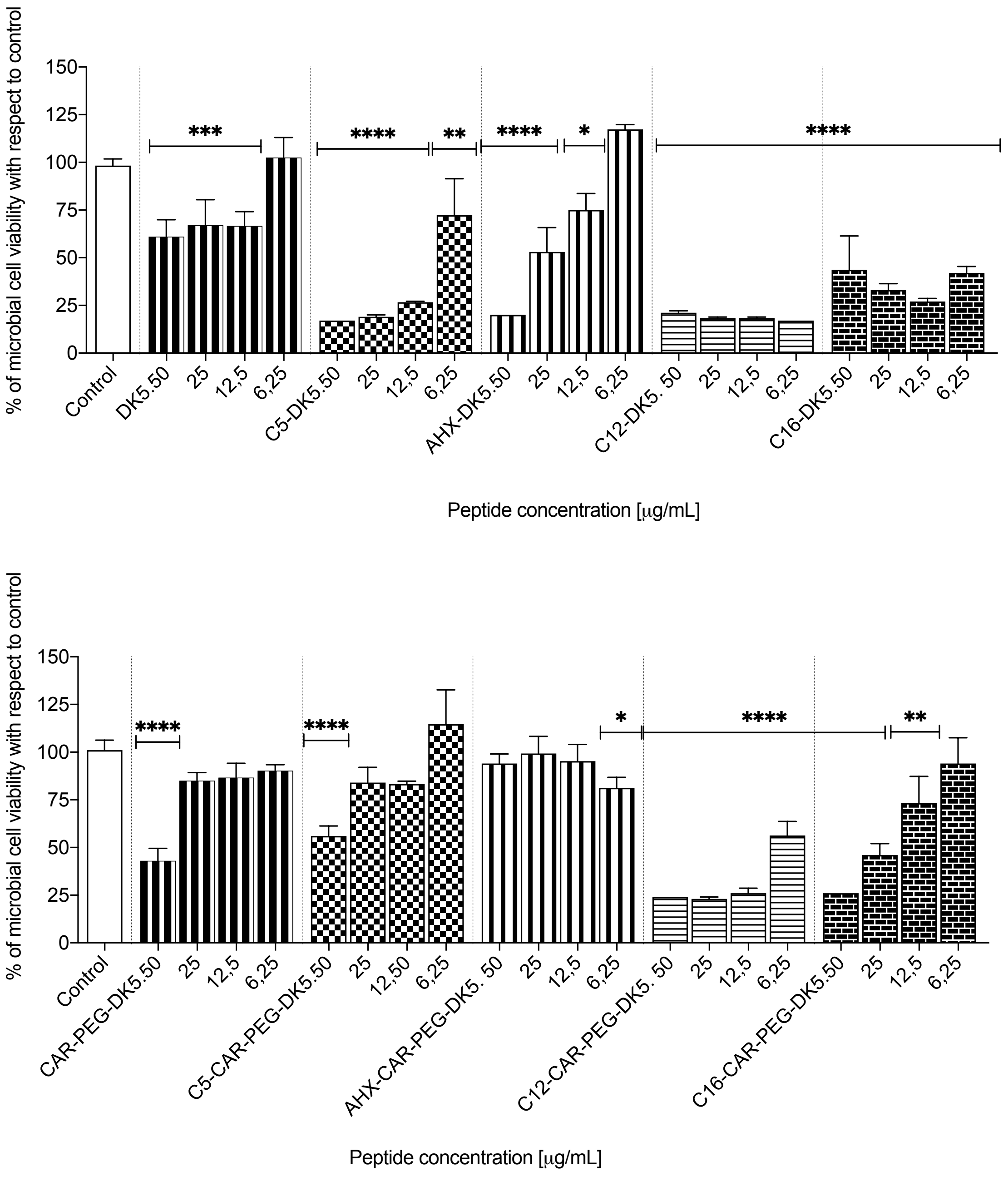

Figure S4. Inhibition of *Candida albicans* biofilm formation by the tested lipopeptides. The antibiofilm activity of the compounds against *C. albicans* (CCM 8186) strain was determined after 24 hours of incubation of the microorganisms in the presence of the selected lipopeptides. The data represents results of MTT assay performed at the end of the experiment. Microbial cell viability within pre-formed biofilms is expressed as the percentage of the cell viability of certain probes with respect to control (microorganisms incubated without peptides). The results were obtained from three independent experiments performed in triplicates and are expressed with standard deviation (error bars). *P < 0.01, **P < 0.005, ***P < 0.0005, ****P < 0.0001 vs. control

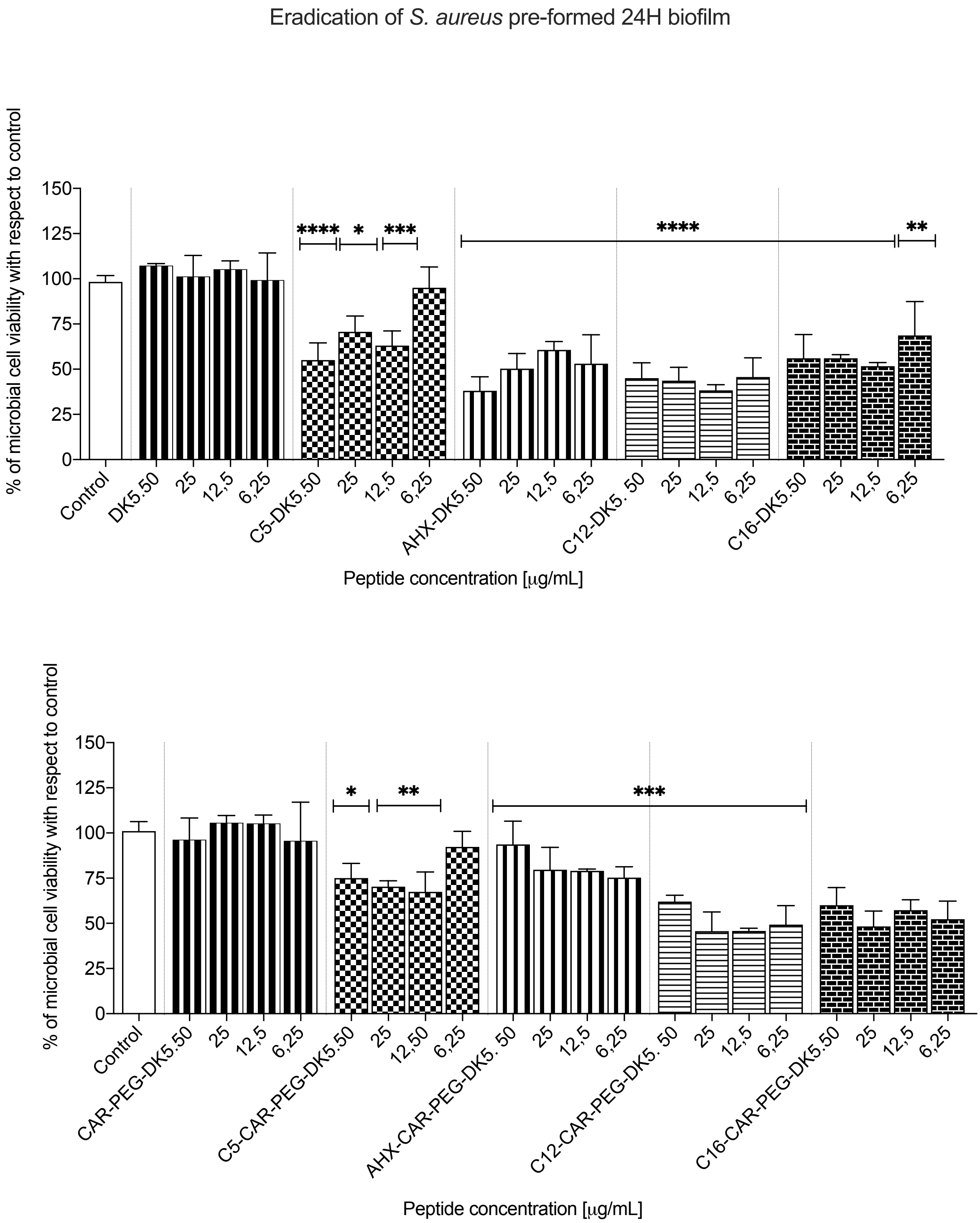

Figure S5. Eradication of the pre-formed 24-hour old biofilm of *S. aureus* after 24-hour treatment with the tested lipopeptides. The antibiofilm activity of the compounds against pre-matured *S. aureus* (PCM 2054) biofilm was determined after 24 hours of incubation of the microorganisms in the presence of the selected lipopeptides. The data represents results of MTT assay performed at the end of the experiment. Microbial cell viability within pre-formed biofilms is expressed as the percentage of the cell viability of certain probes with respect to control (microorganisms incubated without peptides). The results were obtained from three independent experiments performed in triplicates and are expressed with standard deviation (error bars).* $P < 0.01$, **$P < 0.005$, ***$P < 0.0005$, ****$P < 0.0001$ vs. control

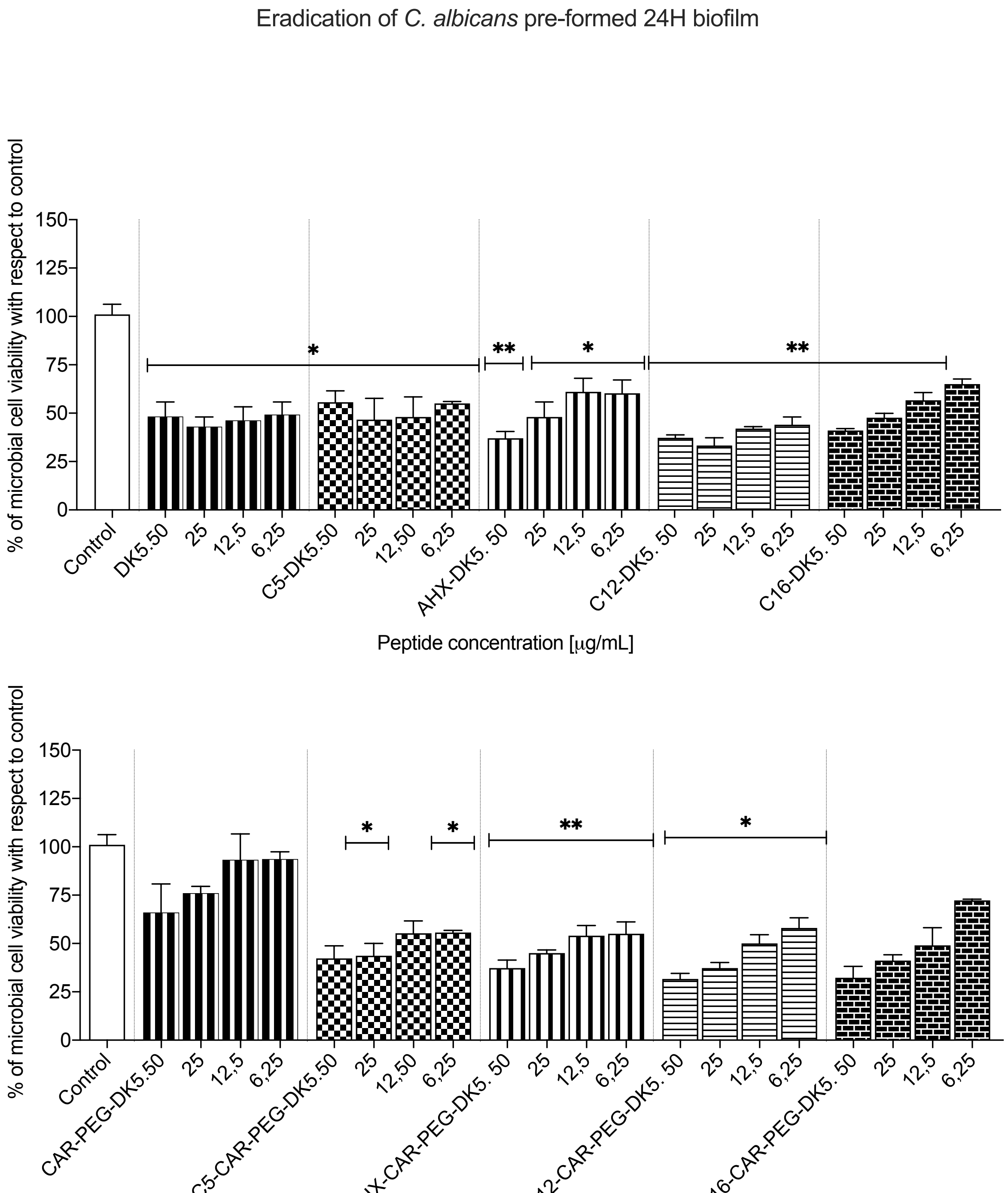

Figure S6. Eradication of the pre-formed 24-hour old biofilm of *C. albicans* after 24-hour treatment with the tested lipopeptides. The antibiofilm activity of the compounds against pre-matured *C. albicans* (CCM 8186) biofilm was determined after 24 hours of incubation of the microorganisms in the presence of the selected lipopeptides. The data represents results of MTT assay performed at the end of the experiment. Microbial cell viability within pre-formed biofilms is expressed as the percentage of the cell viability of certain probes with respect to control (microorganisms incubated without peptides). The were obtained from two independent experiments performed in triplicates and are expressed with standard deviation (error bars).* $P < 0.01$, **$P < 0.005$ vs. control
